# Mechanism of LEF1-AS1 regulating HUVEC cells by targeting miR-489-3p/S100A11 axis

**DOI:** 10.7717/peerj.16128

**Published:** 2023-11-01

**Authors:** Haoran Zhang, Wenqiu Wang, Junjie Lin, Junbo Qiao, Xinjun Wang, Bin Fang, Changkuan Chen, Yujiao Wang, Gaozan Zhu, Wenbo Liu

**Affiliations:** Hemangioma Surgery Department, The Third Affiliated Hospital of Zhengzhou University, Zhengzhou, Henan, China

**Keywords:** Venous malformations, LEF1-AS1, miR-489-3p, S100A11, HUVEC

## Abstract

**Background:**

The venous malformation is the most common congenital vascular malformation and exhibits the characteristics of local invasion and lifelong progressive development. Long noncoding RNA (lncRNA) regulates endothelial cells, vascular smooth muscle cells, macrophages, vascular inflammation, and metabolism and also affects the development of venous malformations. This study aimed to elucidate the role of the lncRNA LEF1-AS1 in the development of venous malformations and examine the interaction among LEF1-AS1, miR-489-3p, and S100A11 in HUVEC cells.

**Methods:**

Venous malformation tissues, corresponding normal venous tissues, and HUVEC cells were used. Agilent human lncRNA microarray gene chip was used to screen differential genes, RNA expression was detected using quantitative reverse transcription PCR, and protein expression was detected using Western blotting. The proliferation, migration, and angiogenesis of HUVEC cells were assessed using CCK8, transwell, and *in vitro* angiogenesis tests.

**Results:**

A total of 1,651 lncRNAs were screened using gene chip analysis, of which 1015 were upregulated and 636 were downregulated. The lncRNA LEF1-AS1 was upregulated with an obvious difference multiple, and the fold-change value was 11.03273. The results of the analysis performed using the StarBase bioinformatics prediction website showed that LEF1-AS1 and miR-489-3p possessed complementary binding sites and that miR-489-3p and S100A11 also had complementary binding sites. The findings of tissue experiments revealed that the expressions of LEF1-AS1 and S100A11 were higher in tissues with venous malformations than in normal tissues, whereas the expression of miR-489-3p was lower in venous malformations than in normal tissues. Cell culture experiments indicated that LEF1-AS1 promoted the proliferation, migration, and angiogenesis of HUVEC cells. In these cells, LEF1-AS1 targeted miR-489-3p, which in turn targeted S100A11. LEF1-AS1 acted as a competitive endogenous RNA and promoted the expression of S100A11 by competitively binding to miR-489-3p and enhancing the proliferation, migration, and angiogenesis of HUVEC cells. Thus, LEF1-AS1 participated in the occurrence and development of venous malformation.

**Conclusions:**

The expression of LEF1-AS1 was upregulated in venous malformations, and the expression of S100A11 was increased by the adsorption of miR-489-3p to venous endothelial cells, thus enhancing the proliferation, migration, and angiogenesis of HUVEC cells. In conclusion, LEF1-AS1 is involved in the occurrence and development of venous malformations by regulating the miR-489-3p/S100A11 axis, which provides valuable insights into the pathogenesis of this disease and opens new avenues for its treatment.

## Introduction

Venous malformations (VMs) are the most common congenital vascular malformations (CVMs). VMs often cause venous structural malformation owing to abnormal vein development after birth and continue to develop with the growth and development of the body (Lee, 2013). VMs lead to disordered angiogenesis and proliferation of endothelial cells and smooth muscle cells, which is characterized by the gradual expansion and tortuous clustering of malformed veins with growth and development (Hage et al., 2018). As a low-flow vascular malformation, VMs are similar to sponges in morphology and structure and comprise endothelial cells and numerous blood sinuses of varying sizes and shapes. Blood coagulation in the sinus cavity can result in thrombus formation, which can be calcified into a phlebolith. The formation of the thrombus may cause inflammatory reactions and swelling, which affect adjacent structures and cause pain (Olivieri et al., 2016). Despite the progress in the clinical treatment of VMs, the main goal of the current therapeutic approach is to reduce symptoms and not eradicate the disease (Dasgupta & Patel, 2014). The intervention effect of most treatment methods is still not ideal. Therefore, further research is required to establish a better therapeutic scheme and improve the clinical results. Our microarray differential screening results show that the long noncoding RNA (lncRNA) LEF1-AS1 promotes the development of VMs. In this study, differentially expressed lncRNAs associated with VMs were screened using the gene chip technology, of which the differentially expressed multiple of LEF1-AS1 was the highest. In addition, recent studies have shown that LEF1-AS1 is highly expressed in liver cancer tissues and cells and that its upregulation leads to a significant increase in hepatocyte proliferation, migration, and invasion as well as angiogenesis of human umbilical vein endothelial cells (Dong et al., 2020). Other studies have reported that LEF1-AS1 regulates the proliferation and migration of vascular smooth muscle cells by targeting the miR-544a/PTEN axis (Zhang et al., 2019b). These results signify that LEF1-AS1 plays a key role in regulating vascular function. However, its role in the occurrence and development of VMs has not been explored so far.

LEF1-AS1 is a recently discovered lncRNA, which is lowly expressed in myeloid malignancies but highly expressed in glioblastoma, lung cancer, hepatocellular carcinoma, osteosarcoma, colorectal cancer, oral squamous cell carcinoma, prostate cancer, retinoblastoma (Cheng et al., 2020a, 2020b; Congrains-Castillo et al., 2019; Gao et al., 2020; He & Qin, 2020; Li et al., 2020; Lu, Qiao & Liu, 2020; Xiang et al., 2020; Zhang et al., 2019a), and other malignancies. The abnormal expression of LEF1-AS1 is closely related to tumorigenesis, development, survival, and prognosis *via* the regulation of target genes and signaling pathways. MicroRNA (miRNA) binding prediction for LEF1-AS1 was performed in this study using the StarBase database, and the results indicated that LEF1-AS1 might target miR-489-3p. Moreover, a previous study has shown that LEF1-AS1 targets miR-489-3p in glioma cells (Cheng et al., 2020a). Another study has reported that ginsenoside Rg1 increases Sirt3 expression by promoting the downregulation of miR-489-3p, which enhances angiogenesis and alleviates diabetic foot ulcers (Huang et al., 2021). Thus, it is evident that miR-489-3p plays an important role in the regulation of vascular function, but its role in the occurrence and development of VMs and its interaction with miR-489-3p in HUVEC cells have not been determined. Hence, miR-489-3p was selected as the research object. The StarBase database was used to predict the mRNA binding of miR-489-3p, and the results showed that miR-489-3p and S100A11 had complementary binding sites. Some studies have observed that the increased expression of S100A11 in EIF3C exosomes promotes angiogenesis and both EIF3C and S100A11 are highly expressed in liver cancer, and their protein levels are associated with the low survival rate of patients with liver cancer (Lee et al., 2018). According to this previous study of Lee, S100A11 plays a certain role in the regulation of vascular function. Furthermore, S100A11 was screened in the mRNA database of our VM gene chip. Hence, it was selected as the research object. Whether LEF1-AS1 is abnormally expressed in VMs is unclear; moreover, the regulatory mechanism of the ceRNA network comprising LEF1-AS1, miR-489-3p, and S100A11 in HUVEC cells has not been studied. In this study, LEF1-AS1 was shown to promote the development of VMs by adjusting the miR-489-3p/S100A11 axis. Our findings provide valuable support for the use of LEF1-AS1 as a potential target in the treatment of VMs and lay the foundation for its use as a new drug target in the future.

## Materials and Methods

### Ethical statement

This study was approved by the Institutional Review Committee of the Third Affiliated Hospital of Zhengzhou University. Each participant provided written informed consent. The scheme was approved by the Scientific Research Ethics Committee of the Third Affiliated Hospital of Zhengzhou University ([2020] YLS No. 119).

### Bioinformatics analysis

The target genes of miRs and miR-489-3p regulated downstream of lncRNA LEF1-AS1 were predicted using the StarBase database (http://starbase.sysu.edu.cn/index.php), an interactive analysis database of gene expression profiles.

### Research objects

From January 2021 to March 2022, VM tissues and adjacent normal venous tissues were obtained from 38 patients diagnosed with VMs and treated surgically in the Third Affiliated Hospital of Zhengzhou University. Fresh tissues were temporarily placed in liquid nitrogen and stored permanently at −80 °C. Inclusion criteria for the experimental group were as follows: (1) VM was confirmed *via* clinical and pathological diagnosis; (2) surgical resection was performed without sclerosing agent injection, laser, interventional embolization, targeted drugs, or other treatments before the operation; (3) no basic diseases of important organs; (4) no special infections, such as hepatitis B, hepatitis C, syphilis, and AIDS. Inclusion criteria for the control group were as follows: (1) the incisal margin of the diseased tissue was confirmed as normal venous tissue *via* pathology; (2) the distance between the tissue taken and the cutting edge of the pathologically proven tissue was at least 3 cm; (3) other tissues were the same as those of the experimental group. The following were the exclusion criteria: (1) diagnosis of vascular malformations and vascular malformation syndrome other than VMs; (2) patients with autoimmune diseases; (3) patients with liver and kidney dysfunction; (4) medical records not well preserved and no follow-up record.

### Cell culture and treatment

HUVECs (American Type Culture Collection, ATCC) cells were cultured in ECM (ScienCell, Carlsbad, CA, USA), which is a special endothelial cell culture medium containing 5% serum, and then placed in 37 °C, 5% CO_2_, 95% humidity incubator (Thermo, Waltham, MA, USA). HUVECs in the logarithmic growth period were used to prepare the cell suspension and inoculated into a 6-well culture plate at the density of 1 × 10^5^ cells/well. After culturing for 24 h, the wells were randomly assigned to the NC, si-LEF1-AS1, miR inhibitor, and si lnc+miR inhibitor groups. Transfection was performed in accordance with the instructions of Lipofectamine3000 (Invitrogen, Waltham, MA, USA). After incubation for 6 h in an incubator at 37 °C under 5% CO_2_ and 95% humidity, the transfection medium was replaced with a complete medium. The transfection was continued for 36 h, followed by the subsequent tests. The sequence of the primers used was as follows: NC justice chain: 5′-UUCUCCGAACGUGUCGUTT-3′, antisense chain: 5′-ACGUGACACGUUCGGAGAATT-3′; Si-LEF1-AS1 justice chain: 5′-ACAUUUCGUUUCUAGCCC-3′, antisense chain: 5′-GCUAGAAACGAAAUGUGA-3′. These were synthesized by Huzhou Hippo Biotechnology Co., Ltd., Huzhou, China.

### Gene chip experiment

Agilent human lncRNA microarray 2019 (4 × 180 k, Design ID: 086188) chip was used to detect the samples. After sample quality control, data were standardized using the quantile algorithm, and the differential genes were subsequently screened.

(1) The total RNA of the sample was quantified using NanoDrop ND-2000, and RNA integrity was checked. After quantification of the RNA, sample labeling, hybridization, and chip elution were performed according to the standard process of the chip. First, the total RNA was reverse-transcribed into double-stranded complementary DNA (cDNA), and the cRNA was labeled with fluorescence using cyanine-3-CTP (Cy3). The labeled cRNA was hybridized with the gene chip, and the scanning results were obtained using Agilent Human lncRNA Microarray 2019 (4 * 180 k, Design ID:086188) after elution.

(2) Feature extraction software was adopted to process the scanning result map. To eliminate the data differences between experiments, the quantile method was used for the standardized processing of the original data. Before performing the subsequent analysis, standardized data were screened and filtered. For comparison, in each group of samples, at least 75% of the samples labeled as “detected” were left for follow-up analysis. Differential gene screening was performed using the *P*-value and the multiple change value of the t-test. The screening criteria were upregulated or downregulated with multiple change values of ≥2.0 and a *P*-value of ≤0.05.

### Detection of RNA expression using quantitative reverse transcription (qRT)-PCR

The total RNA of tissues and cells was extracted with TRLzol (Beijing Quanshijin Company, Beijing, China), and RNA was concentrated using isopropanol precipitation. The reverse transcription was performed according to the instructions of the ReversTra Ace qPCR RT Kit (Toyobo Company, Osaka, Japan). The RNA was reverse transcribed into cDNA, and qRT-PCR was performed according to the instructions of SYBR Green Realtime PCR Master Mix Kit (Toyobo Company, Osaka, Japan) for qPCR detection. The sequence of primers used for qRT-PCR are shown in Table 1 (Huzhou Hippo Biotechnology Co., Ltd., Huzhou, China). The reaction conditions were as follows: predenaturation at 95 °C for 60 s, denaturation at 95 °C for 15 s, annealing at 60 °C for 15 s, and extension at 72 °C for 45 s for 40 cycles (the PCR instrument was from Roche, Switzerland). The primers were synthesized by Beijing Qingke Technology Co., Ltd. GAPDH and U6 were used as reference genes to compare the relative cycle threshold (Ct) values of LEF1-AS1, miR-489-3p, and S100A11. The relative expression of the target gene was calculated using the 2^−ΔΔCt^ method; three duplicate were set in each group, and each experiment was repeated thrice.

**Table 1:** Primers for qRT-PCR experiment.

LEF1-AS1	Upstream primer	5′-TAAAATGGATGGACTGGGGCTAT-3′
	Downstream primer	5′-GTAACTGGATAAACAATGAGACTAACGA-3′
GAPDH	Upstream primer	5′-GGAGTCCACTGGCGTCTTCA-3′
	Downstream primer	5′-GTCATGAGTCCTTCCACGATACC-3′
S100A11	Upstream primer	5′-CCAGAAGTATGCTGGAAAGGATG-3′
	Downstream primer	5′-CATCATGCGGTCAAGGACACCA-3′
miR-489-3p	Upstream primer	5′-GCGCGGTGATCATATAC-3′
	Downstream primer	5′-AGTGGGTCGAGTATAT-3′
U6	Upstream primer	5′-CTCGCTTCGGCAGCACA-3′
	Downstream primer	5′-AACGCTTCACGAATTTGCGT-3′

### Western blotting of protein expression

The total protein of tissues and cells was extracted using the BCA protein (Shanghai Biyuntian Biotechnology Co., Ltd., Shanghai, China) quantitative kit to detect the protein concentration and stored at −20 °C as a standby. Glyceraldehyde 3-phosphate dehydrogenase (GAPDH, American Abcam Company, ab9485, working concentration 1:1,000) and S100A11 antibody (American Abcam Company, ab169530, working concentration 1:1,000) were purchased. Subsequently, 15% SDS-PAGE gel (Wuhan Saiwei Biotechnology Co., Ltd., Wuhan, China) was prepared, and 40 µg of the protein was added to each well. Gel electrophoresis was performed to separate the protein, and it was transferred to a polyvinylidene difluoride (PVDF) membrane (membrane transfer condition: 300 mA, 150 min). The membrane was washed with Tris-buffered saline with 0.1% Tween 20 (TBST) three times, 10 min/time. The PVDF membrane was sealed with a rapid sealing solution for 1 h, washed with TBST three times, and strips corresponding to S100A11 and GAPDH were cut. The corresponding primary antibody was added and incubated at 4 °C overnight. The next day, the film was initially washed with TBST three times, 10 min/time, and then a fluorescent secondary antibody (Odyssey Company, Stamford, CT, USA, working concentration 1:10,000) was added and incubated in a dark shaking bed for 1 h. The film was washed with TBST buffer three times, 10 min/time, and finally detected using a protein imprint exposure imager. Image J software was used to measure the band gray value, the relative protein expression was calculated, and GraphPad Prism 9.0 was used to construct a histogram.

### Double luciferase reporter gene assay

(1) The possible binding sites of miR-489-3p and LEF1-AS1 as well as those of miR-489-3p and S100A11 were predicted by searching the bioinformatics website StarBase database. The LEF1-AS1 3′-UTR sequences containing the binding sites of miR-489-3p were obtained. The 3′-UTR sequence of S100A11 and that of the mutant (Mut) (Huzhou Hippo Biotechnology Co., Ltd., Beijing, Chain.) synthesized using the chemical method with the corresponding binding sites were cloned into the vector.

(2) HUVEC cells were coated in 12-well plates and cotransfected with WT or Mut type and miR-489-3p mimic and Lipo3000 (Invitrogen, the United States) in HUVEC cells when the cell confluence reached 60–80%.

(3) Forty-eight hours after transfection, firefly luciferase (Fluc) and sea luciferase (Rluc) activities in each group were detected using the reporter luciferase assay kit (Dual-Luciferase Reporter Assays, Promega), and the relative expressions of each reporter gene were expressed using Fluc/Rluc values.

(4) Statistical analysis was performed to determine whether there were statistically significant differences between the groups.

### Detection of cell proliferation using the CCK-8 assay

Each group of cells was inoculated into 96-well plates according to the standard of 100 µL of cell culture medium and 3000 cells in each well. The cells were incubated at 37 °C in a 5% CO_2_ incubator for 24, 48, and 72 h, following which the supernatant was discarded. Subsequently, 100 µL of cell culture solution and 10 µL of CCK-8 solution were added (Toyo Textile Company, Tokyo, Japan). These solutions were mixed evenly and incubated for 2 h. Finally, the optical density of each well was measured at a wavelength of 450 nm with an automatic microplate reader.

### Detection of cell migration using the transwell assay

In the upper chamber of the transwell plate (Corning Company, New York, NY, USA), HUVEC cells transfected for 36 h were added and resuspended in 200 µL (2 × 10^4^ cells/well) of serum-free medium. Later, 600 µL of RPMI1640 medium containing 20% fetal bovine serum was added to the 24-well plate (Beijing Solebar Technology Co., Ltd., Beijing, China), and the cells were added. Each group of cells was set with three reperforators and placed in an incubator at 37 °C with 5% CO_2_ and 95% humidity for 36 h. The chamber was removed, the liquid in the upper chamber was sucked out, rinsed gently with phosphate-buffered saline (PBS), fixed with 4% methanol for 20 min, stained with crystal violet for 10 min, rinsed twice with PBS, the residual cells in the upper chamber were gently wiped off with a cotton swab, and the cells migrated to the lower layer of the microporous membrane were counted under an inverted microscope. The ImageJ software was used for statistical analysis.

### Angiogenesis experiment

The matrix adhesive (BD Company, Franklin Lakes, NJ, USA) was added to the 24-well plate with a capacity of 300 µL/hole for solidification. The HUVEC cells were resuspended in ECM culture medium, inoculated on a Matrigel-coated 24-well plate at a density of 8 × 10^4^ cells/well, and incubated at 37 °C for 6 h. Each group of cells was set with three multiple wells. The growth was observed under an inverted microscope, and the tube formation of HUVEC cells was photographed. The ImageJ software was used to count the length of the assembly tube.

### Statistical analysis

SPSS26.0 and GraphPad Prism 8.0 software were used for statistical analysis. Independent samples *t*-test was used for comparison between two groups, and one-way analysis of variance was used for comparison between multiple groups. The difference was considered statistically significant if *P* < 0.05.

## Results

### LncRNA expression profile in VMs

**Figure 1:**
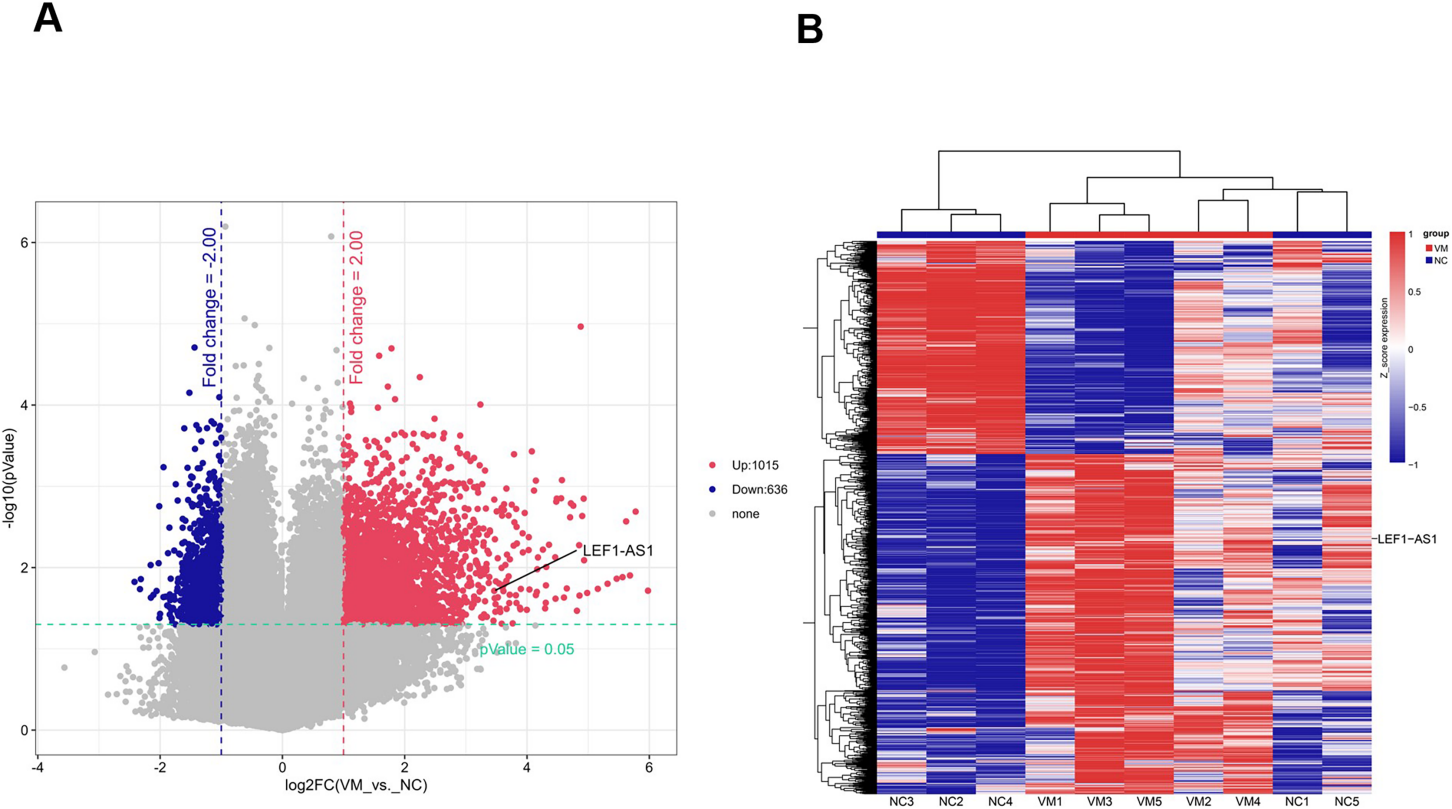
Screened candidate lncRNA biomarkers through chip and bioinformatics analyses. (A) The differentially expressed lncRNAs were selected by volcanic map, filtering two vertical lines were the double change boundary, and the horizontal line indicates the statistically significant boundary (*P* < 0.05), the upstream and downstream genes with multiple changes ≥2 and statistical significance are marked with red dots and blue dots, respectively. (B) The cluster diagram according to the color code description of relative lncRNA expression, red represents upregulation, blue represents downregulation, and the numbers 0.8, 0, and −0.8 indicate the multiple changes in the corresponding spectrum.

To identify lncRNAs that are preferentially expressed in human VM tissues and possibly contribute to the progression of the disease, candidate lncRNA biomarkers were screened using chip and bioinformatics analysis. The expression profiles of differentially expressed lncRNAs in VM tissues and their paired normal tissues were evaluated. These lncRNAs were from five patients with VMs. The Agilent human lncRNA microarray was used, and the differentially expressed lncRNAs were identified by performing microarray analysis. The analysis detected 1651 lncRNAs that were differentially expressed in VMs, with at least twice the change. Of these, 1015 lncRNAs were upregulated and 636 were downregulated. The differentially expressed lncRNAs were selected with volcanic map filtering (fold change ≥ 2 and *P*-value ≤ 0.05). As depicted in the volcanic map (Fig. 1A), the two vertical lines represented the double change boundary and the horizontal line denoted the statistically significant boundary (*P* < 0.05). The upregulated and downregulated genes with multiple changes ≥2 and statistical significance were marked with red dots and blue dots, respectively. Candidate lncRNA biomarkers were selected based on the results of microarray and bioinformatics analyses. The microarray results showed that the NC sample was the normal tissue of the patient. Compared with normal vascular tissues, multiple lncRNAs were abnormally expressed in VMs. The cluster diagram of the lncRNA profile comparison between VMs and paired normal venous vascular samples is shown in Fig. 1B. In the figure, each row represents an lncRNA and each column represents a tissue sample. According to the color code description of relative lncRNA expression, red represents upregulation, blue represents downregulation, and the numbers 0.8, 0, and −0.8 signify multiple changes in the corresponding spectrum. The hierarchical clustering of the expressions of 1651 lncRNAs clearly distinguished VMs from normal tissues.

### The lncRNA LEF1-AS1-miR-489-3p-S100A11 interaction network

**Figure 2:**
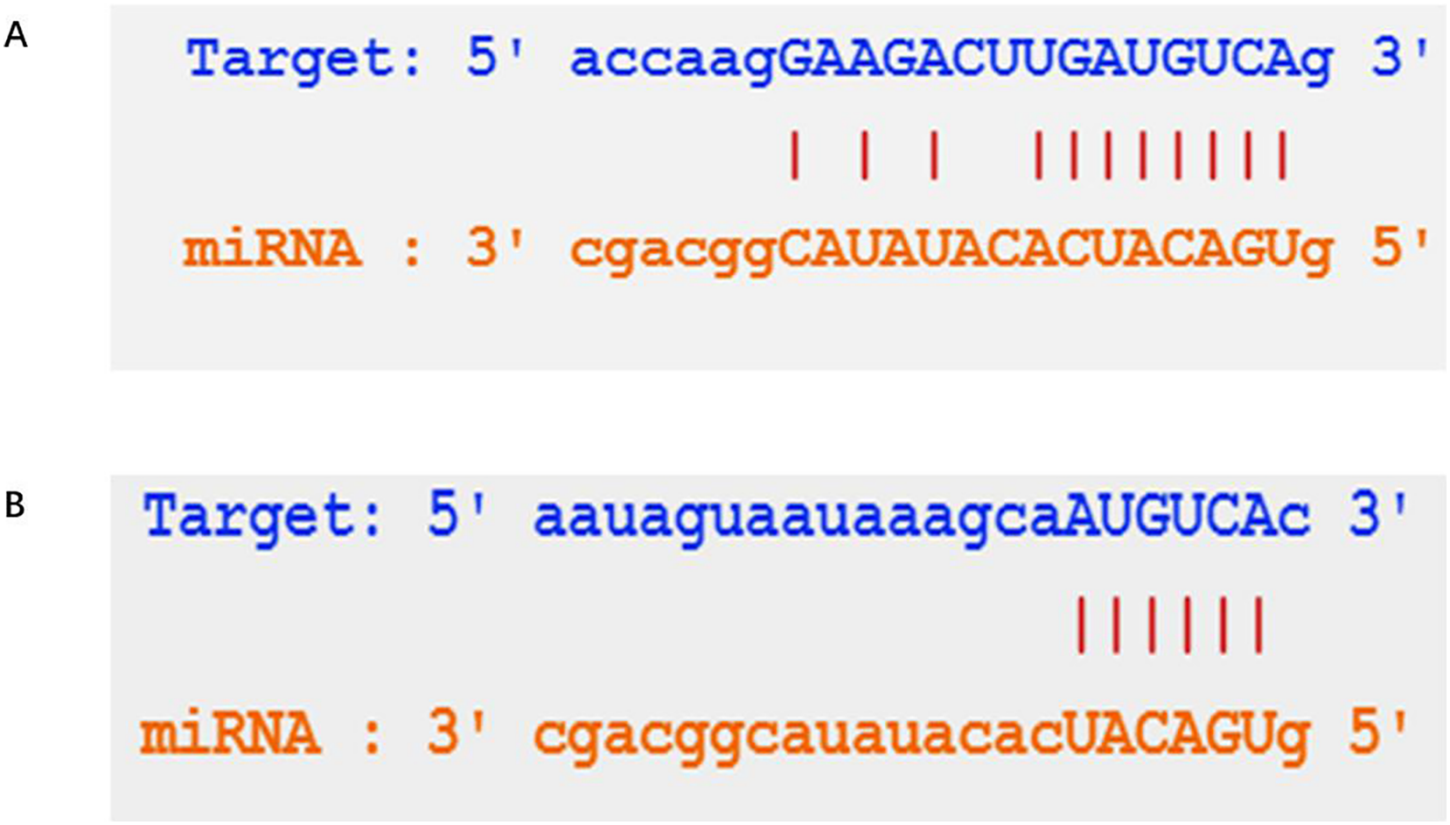
Prediction of the downstream in the StarBase database. (A) Prediction of the targets of LEF1-AS1. (B) Prediction of the targets of miR-489-3p.

This study aimed to determine the role of the lncRNA LEF1-AS1 in VMs. The downstream regulatory miRs of LEF1-AS1 were predicted using the StarBase online prediction website, and the binding sites of LEF1-AS1 and miR-489-3p (Fig. 2A) were obtained. The upper sequence was LEF1-AS1, and the lower sequence was miR-489-3p. The downstream regulatory genes of miR-489-3p were explored in the StarBase database, and the binding sites of miR-489-3p and S100A11 were obtained (Fig. 2B). The upper sequence was S100A11, and the lower sequence was miR-489-3p. The findings revealed that the lncRNA LEF1-AS1-miR-489-3p-S100A11 axis may be involved in the occurrence and development of VMs.

### The high expression of lncRNAs LEF1-AS1 and S100A11 and the low expression of miR-489-3p in VMs

**Figure 3:**
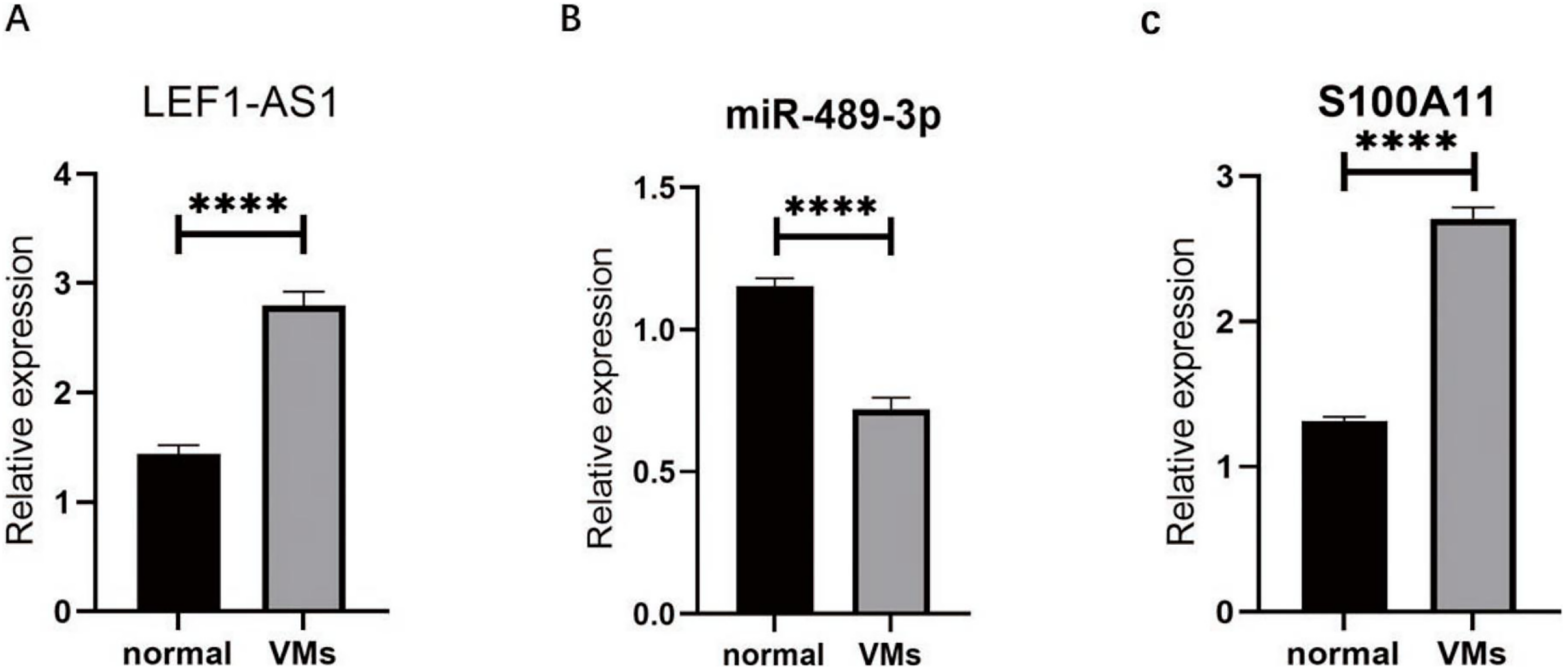
(A) LEF1-AS1 was upregulated in venous malformations. (B) miR-489-3p was downregulated in venous malformations. (C) S100A11 was upregulated in venous malformations. ****P < 0.0001.

RT-qPCR was used to detect the VM tissues of 38 patients. The results showed that compared with the expressions of LEF1-AS1, miR-489-3p, and S100A11 in normal veins, the expression of LEF1-AS1 in VM tissues was significantly higher (Fig. 3A, *P* < 0.0001), and the difference was statistically significant. The expression of S100A11 was also significantly increased (Fig. 3C, *P* < 0.0001). On the contrary, the expression of miR-489-3p decreased significantly (Fig. 3B, *P* < 0.0001), and the difference was statistically significant.

### Regulation of the expression of S100A11 by LEF1-AS1 *via* adsorption onto miR-489-3p

**Figure 4:**
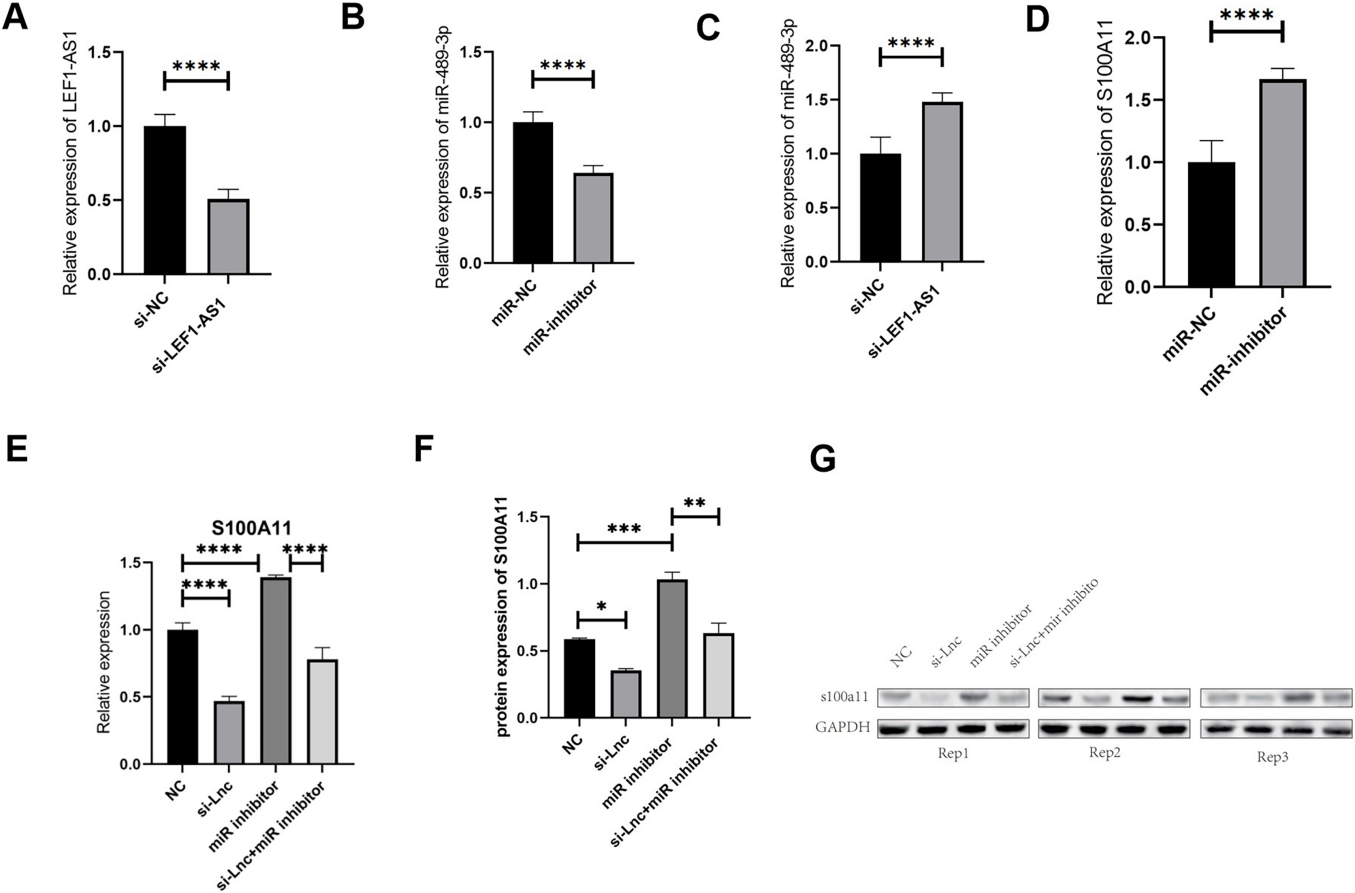
(A) The relative expression of LEF1-AS1 in the si-NC group and si-LEF1-AS1 groups, (B) the relative expression of miR-489-3p in the miR-NC group and Mir-inhibitor groups, (C) the relative expression of miR-489-3p in the si-NC and si-LEF1-AS1 groups, LEF1-AS1 negatively regulated the expression of miR-489-3p. (D) The relative expression of S100A11 in the miR-NC and Mir-inhibitor groups, miR-489-3p negatively regulated the expression of S100A11. (E) PCR results showed that LEF1-AS1 silencing inhibited the expression of S100A11, which could be covered by a miR-489-3p inhibitor. The expression inhibition of miR-489-3p could promote the expression of S100A11, (F) the results of WB showed that LEF1-AS1 silencing inhibited the expression of S100A11; this inhibitory effect could be covered by a miR-489-3p inhibitor. The expression inhibition of miR-489-3p could promote the expression of S100A11, *P < 0.05, **P < 0.01, ***P < 0.001, ****P < 0.0001. (G) The relative expression of S100A11 protein in the four groups: NC, si-LEF1-AS1, miR inhibitor, and si-LEF1-AS1+miR inhibitor.

The results of the above tissue experiments verified the ceRNA regulatory mechanism of the lncRNA LEF1-AS1, and the findings indicated that LEF1-AS1 may be involved in regulating the occurrence and development of VM. However, the specific mechanism of action needs to be further explored by performing cell culture experiments. After transfection of HUVEC cells, whether LEF1-AS1 and miR-489-3p were silenced successfully was determined using qRT-PCR assay. The results showed that the relative expression of LEF1-AS1 in the si-LEF1-AS1 group was significantly lower than that in the si-NC group (Fig. 4A, *P* < 0.0001), and the difference was statistically significant, which indicated the successful silencing of LEF1-AS1 in HUVEC cells. HUVEC can be used for subsequent experiments. The relative expression of miR-489-3p in the miR-inhibitor group was significantly lower than that in the miR-NC group (Fig. 4B, *P* < 0.0001), and the difference was statistically significant. This result indicated that the expression of miR-489-3p was successfully inhibited and that these cells could be used for subsequent experiments. Bioinformatics prediction results showed that LEF1-AS1 and miR-489-3p possessed complementary binding sites, and qRT-PCR results showed that LEF1-AS1 silencing increased the expression of miR-489-3p in HUVEC cells (Fig. 4C, *P* < 0.0001). This observation indicated that miR-489-3p interacted with LEF1-AS1, which in turn negatively regulated the expression of miR-489-3p. Furthermore, bioinformatics prediction results demonstrated the presence of a binding site between the miR-489-3p sequence and the 3′UTR region of S100A11. In addition, qRT-PCR detection results showed that inhibiting the expression of miR-489-3p significantly increased the expression of S100A11 (Fig. 4D, *P* < s0.0001). These results signified that the binding sites of miR-489-3p and S100A11 were bound to each other and negatively correlated. The ceRNA mechanism of LEF1-AS1 in HUVEC cells was further confirmed by performing a rescue experiment. The findings showed that a single transfection with si-LEF1-AS1 reduced the expression of S100A11, whereas a single transfection with miR-489-3p inhibitor increased the expression of S100A11. Cotransfection with si-LEF1-AS1 and miR-489-3p inhibitor partially reversed the inhibition of S100A11 expression caused by LEF1-AS1 silencing. The outcomes of qRT-PCR and Western blotting implied that the inhibition of LEF1-AS1 expression reduced the expression of S100A11 at both mRNA and protein levels and that the inhibition was negatively regulated by miR-489-3p (Figs. 4E–4G). In conclusion, LEF1-AS1 can regulate the expression of S100A11 by competitively binding to miR-489-3p.

### The targeting of miR-489-3p by LEF1-AS1, and S100A11 acting as the target gene of miR-489-3p

**Figure 5:**
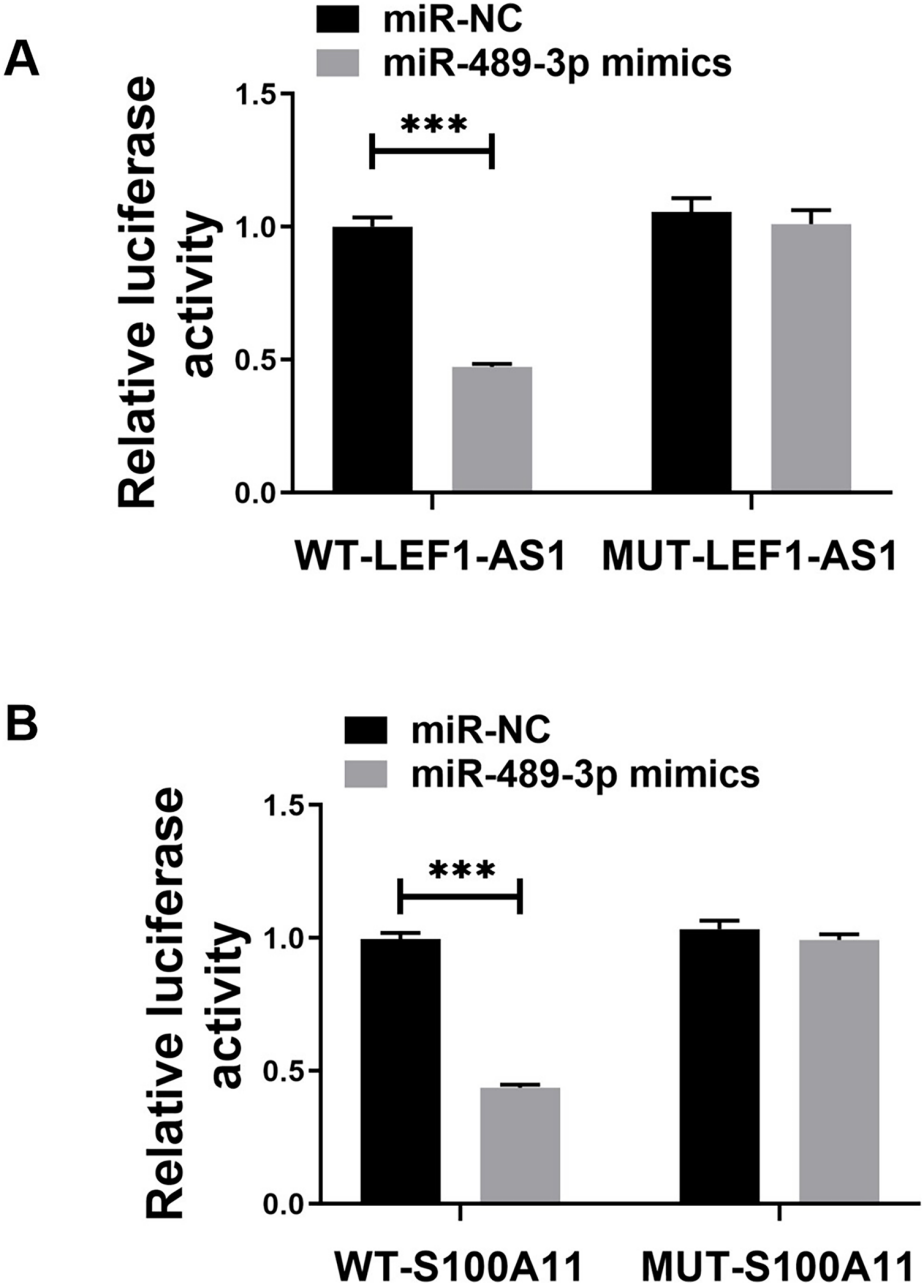
(A) Dual luciferase analysis showing that LEF1-AS1 could target miR-489-3p, (B) dual luciferase analysis showed that S100A11 could target miR-489-3p in HUVEC, ***P < 0.001.

The above bioinformatics prediction results revealed the possible presence of complementary binding sites between LEF1-AS1 and miR-489-3p. Furthermore, there were possible complementary binding sites between miR-489-3p and S100A11. Meanwhile, the above PCR results denoted that LEF1-AS1 silencing increased the expression of miR-489-3p in HUVEC cells (Fig. 4C, *P* < 0.0001) and that LEF1-AS1 negatively regulated the expression of miR-489-3p. Moreover, the PCR results signified that inhibiting the expression of miR-489-3p enhanced the expression of S100A11 (Fig. 4D, *P* < 0.0001) and that miR-489-3p negatively regulated the expression of S100A11. The results of the double luciferase assay showed that after transfection of the wild-type LEF1-AS1 sequence, the activity of luciferase was significantly inhibited in the miR-489-3p mimic group, but the fluorescence intensity of the mutant plasmid did not change significantly (Fig. 5A, *P* < 0.001). After transfection of the wild-type S100A11 sequence, luciferase activity was significantly inhibited in the Mir-489-3p mimic group. Although the activity of luciferase was significantly inhibited in the miR-489-3p mimic group, the fluorescence intensity of mutant plasmids did not change significantly (Fig. 5B, *P* < 0.001). Based on the above bioinformatics prediction results and those of qRT-PCR, the results of dual luciferase assay better demonstrate that LEF1-AS1 in HUVEC cells can target miR-489-3p and that S100A11 is the target gene of miR-489-3p.

### Effects of LEF1-AS1 on proliferation, migration, and angiogenesis of HUVEC cells *via* the miR-489-3p/S100A11 axis

**Figure 6:**
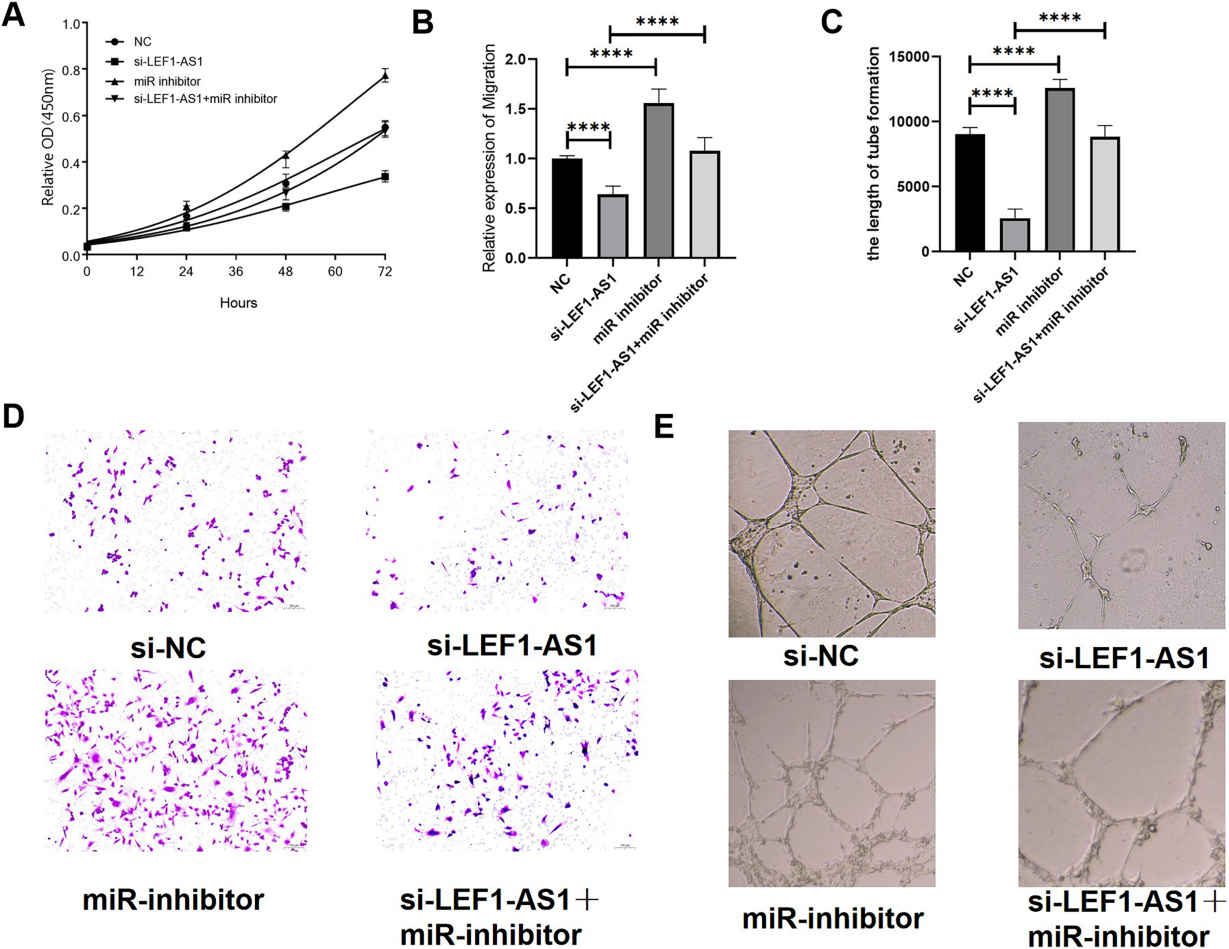
(A) LEF1-AS1 silencing had an inhibitory effect on cell proliferation, which was weakened by miR inhibitor, while the inhibition of Mir-489-3p expression enhanced cell proliferation. (B) LEF1-AS1 silencing had an inhibitory effect on cell migration, which was weakened by a miR inhibitor. The expression inhibition of Mir-489-3p enhanced the cell migration, (C) LEF1-AS1 silencing had an inhibitory effect on cell angiogenesis, which was weakened by a miR inhibitor, while the inhibition of Mir-489-3p expression enhanced the cell angiogenesis, (D) transwell migration maps of the four groups of cells: NC, si-LEF1-AS1, miR inhibitor, and si-LEF1-AS1+miR inhibitor. (E) NC, si-LEF1-AS1, miR inhibitor, and si-LEF1-AS1+miR inhibitor groups, *in vitro* angiogenesis of the four groups of cells. ****P < 0.0001.

The above results confirmed the interaction among the three factors LEF1-AS1, miR-489-3p, and S100A11. In HUVEC cells, LEF1-AS1 regulated the expression of S100A11 by regulating miR-489-3p. Subsequently, the effect of the ceRNA mechanism exerted by LEF1-AS1 on the proliferation, migration, and angiogenesis of HUVECs was examined. After transfection with si-LEF1-AS1 alone, the proliferation and migration as well as *in vitro* angiogenesis of HUVEC cells were decreased. On the contrary, after transfection with miR-489-3p inhibitor alone, the proliferation, migration, and *in vitro* angiogenesis was enhanced. The inhibitory effect of LEF1-AS1 silencing on HUVEC proliferation, migration, and angiogenesis was negatively regulated by miR-489-3p. Cotransfection with si-LEF1-AS1 and miR-489-3p inhibitor partially reversed the proliferation (Fig. 6A), migration (Figs. 6B and 6C), and angiogenesis (Figs. 6D and 6E) of HUVEC cells caused by LEF1-AS1 silencing, and the differences were statistically significant. Combined with the present experimental results, it was concluded that the high expression of LEF1-AS1 in VMs results in the adsorption of miR-489-3p to venous endothelial cells, thereby increasing the expression of S100A11. Consequently, the proliferation, migration, and angiogenesis of HUVEC cells are enhanced. This ceRNA mechanism of action is employed by LEF1-AS1 to promote the development of VMs.

## Discussion

VMs can occur in any part of the body and exhibit the biological characteristics of local invasion. VMs often develop progressively under the influence of hormonal changes, infection, trauma, and other factors. When important organs are involved, dysfunction, bleeding, and even death can occur. There is a high possibility of intraoperative massive hemorrhage during the treatment of multiple severe systemic VM syndrome (Richter & Braswell, 2012). Owing to this risk, patients often lose the chance of radical treatment, and conventional treatment methods have certain drawbacks. Hence, studying the molecular mechanism of VMs is of immense significance for the future radical treatment of multiple severe systemic VM syndrome. Moreover, being the most common CVMs (Legiehn & Heran, 2008), VMs can cause congestion, pain, swelling, functional damage, and disfigurement, and large lesions can cause coagulation disorders. Nonetheless, the treatment options for VMs in molecular and cellular biology remain limited. Mounting evidence suggests that lncRNAs can bind to miRNAs as ceRNAs, thereby affecting and regulating the expression of target genes. LncRNA-miRNA-mRNA ceRNA networks have been reported to play a pertinent role in several types of tumors and other diseases (Wu et al., 2020a). Furthermore, the lncRNA/miRNA interaction can offer new clues for determining diagnostic markers and treating vascular diseases (Zhao et al., 2020). Although VMs are the most common vascular malformations, the role of lncRNA-related ceRNA regulatory mechanisms in VMs is yet to be elucidated. LncRNAs are a group of noncoding RNAs that do not encode proteins. Nevertheless, they can interact with miRNAs to reduce their impact on target mRNA (Xu et al., 2022). LncRNA is also an important regulator of vascular function and is a factor related to angiogenesis. Studies have confirmed that specific lncRNAs play crucial roles in regulating endothelial cells. LncRNAs are involved in the apoptosis, proliferation, and migration of endothelial cells in blood vessels and participate in the injury, self-repair, and formation of new blood vessels in the vascular endothelium (Singh et al., 2016; Teng et al., 2017). VMs are predominantly caused by angiogenesis disorders. Angiogenesis is not static but developing and changing. Alterations in endothelial cell proliferation and migration can drive changes in the vascular structure during angiogenesis, thereby leading to vascular malformations. Therefore, lncRNA may be involved in the development of VMs. To test this hypothesis, in this study, the gene chip technology was used to screen differentially expressed lncRNAs associated with VMs. The findings revealed that LEF1-AS1 had a large differentially expressed multiple and that it might promote the development of VMs. A previous study has observed that LEF1-AS1 can regulate the expression of TGFBR1 by targeting miR-24-3p, which may be a potential therapeutic target for dental pulp bone regeneration (Wu, Lian & Sun, 2020b). LEF1-AS1 is highly expressed in glioblastoma tissues, and in glioblastoma cells, this lncRNA can be targeted to miR-543, leading to the upregulation of EN2 expression. LEF1-AS1/miR-543/EN2 is a novel ceRNA network and is associated with the progression of glioblastoma. These findings provide new insights into the treatment of glioblastoma (Zeng et al., 2020). According to a study, LEF1-AS1 can enhance the expression of PTEN by competitively binding to miR-221, promote the proliferation of non-small cell lung cancer cells and induce cell apoptosis, and thus promote the development of lung cancer (Xiang et al., 2020). In this study, the expression of LEF1-AS1 was found to be upregulated in VM tissues. Furthermore, LEF1-AS1 acted as a sponge on miR-489-3p *via* the ceRNA mechanism, thereby affecting the expression of S100A11 and regulating the proliferation, migration, and angiogenesis of HUVEC cells. In this manner, LEF1-AS1 participated in the occurrence and development of VM. These findings widen our knowledge of the pathogenesis of VMs and offer promising directions and ideas for the treatment.

## Conclusions

Owing to the advancements in clinical research on lncRNA, its functional structure is becoming increasingly clear. This study has examined the relationship between lncRNA and diseases by detecting the expression of lncRNA. Because of its spatiotemporal specificity, lncRNA is of immense significance in disease diagnosis and treatment provides novel ideas for the clinical diagnosis of biomarkers of related diseases, and holds positive significance in identifying new disease regulation mechanisms. LncRNA can provide a new basis as well as suitable targets for the diagnosis and treatment of several complex diseases in the clinic and further aid in understanding the complex regulatory network of higher eukaryotes (Schmitz, Grote & Herrmann, 2016). Our study showed that LEF1-AS1 expression is upregulated in VM tissues. Moreover, LEF1-AS1 can increase the expression of S100A11 by adsorbing onto miR-489-3p in venous endothelial cells, thereby regulating the proliferation, migration, and angiogenesis of HUVEC cells. In conclusion, LEF1-AS1 can participate in the occurrence and development of VMs by regulating the miR-489-3p/S100A11 axis, which provides valuable information on the pathogenesis of VMs and offers potential avenues for therapy. LncRNAs are likely to provide reliable methods for the diagnosis and treatment of VMs and are expected to serve as diagnostic markers or therapeutic targets.

## Supplemental Information

10.7717/peerj.16128/supp-1 Supplemental Information 1 The data of double luciferase experiment.

10.7717/peerj.16128/supp-2 Supplemental Information 2 Analysis of RNA gene chip result.

10.7717/peerj.16128/supp-3 Supplemental Information 3 Raw data for Figures 1–3.

10.7717/peerj.16128/supp-4 Supplemental Information 4 Raw data for Figure 4.

10.7717/peerj.16128/supp-5 Supplemental Information 5 Raw data for Figure 4: Angiogenesis SI.

10.7717/peerj.16128/supp-6 Supplemental Information 6 Raw data for Figure 4: Angiogenesis NC.

## Additional Information and Declarations

### Data Availability

The following information was supplied regarding data availability: The raw measurements are available in the Supplemental Files.
