# Supplementary figures and images for: Mechanism of LEF1-AS1 regulating HUVEC cells by targeting miR-489-3p/S100A11 axis
Source: PeerJ. 2023 Nov 1;11:e16128. doi: 10.7717/peerj.16128 (PMC10625350; doi:10.7717/peerj.16128)

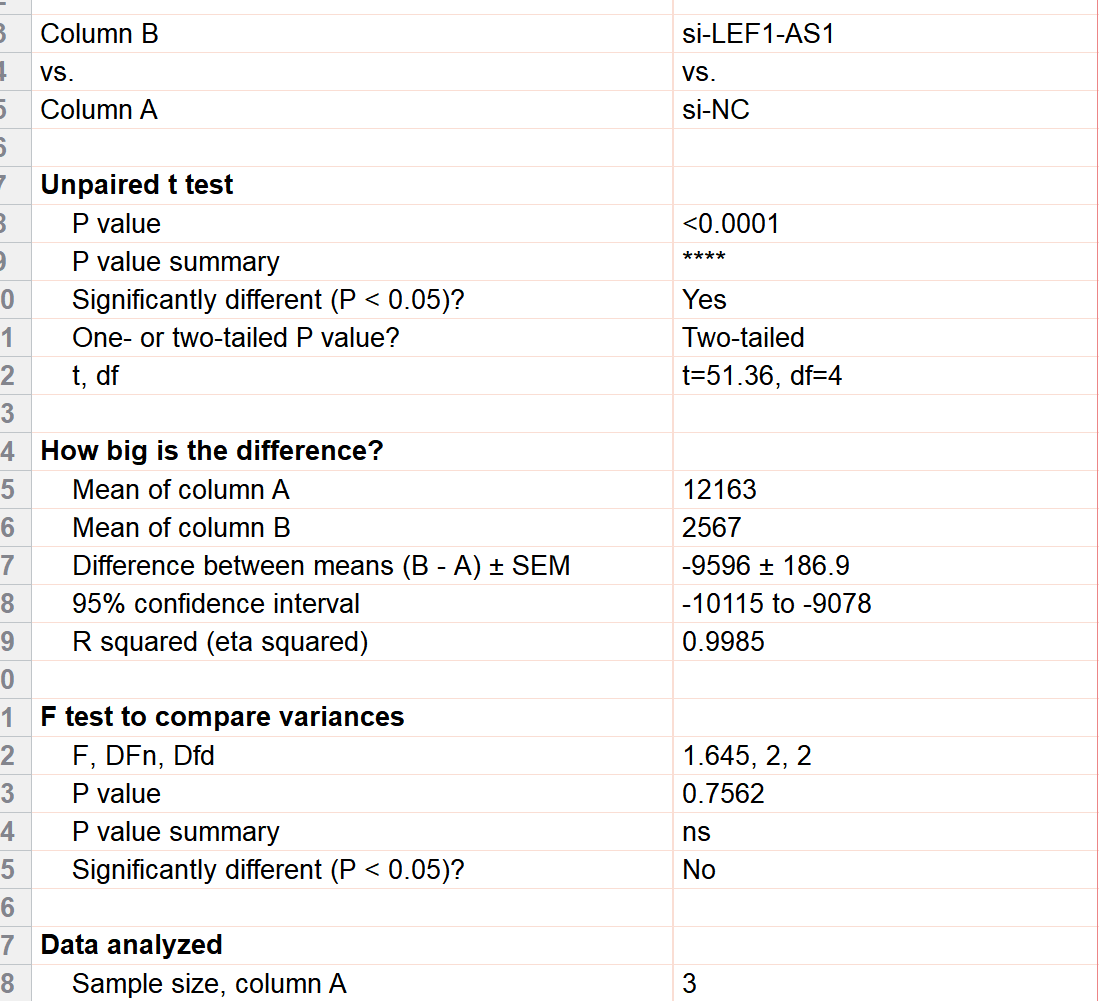

Supplement: Supplemental Information 4 [file peerj-11-16128-s004.zip › date Fig 4/Fig4 date/Angiogenesis.png]

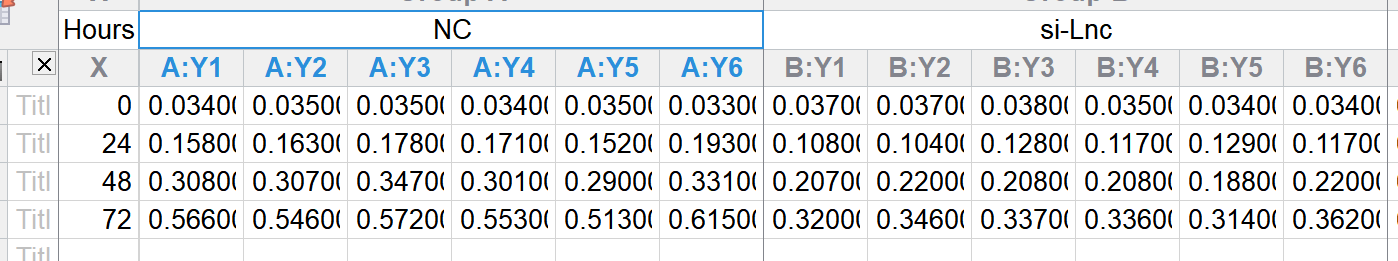

Supplement: Supplemental Information 4 [file peerj-11-16128-s004.zip › date Fig 4/Fig4 date/CCK8 1.png]

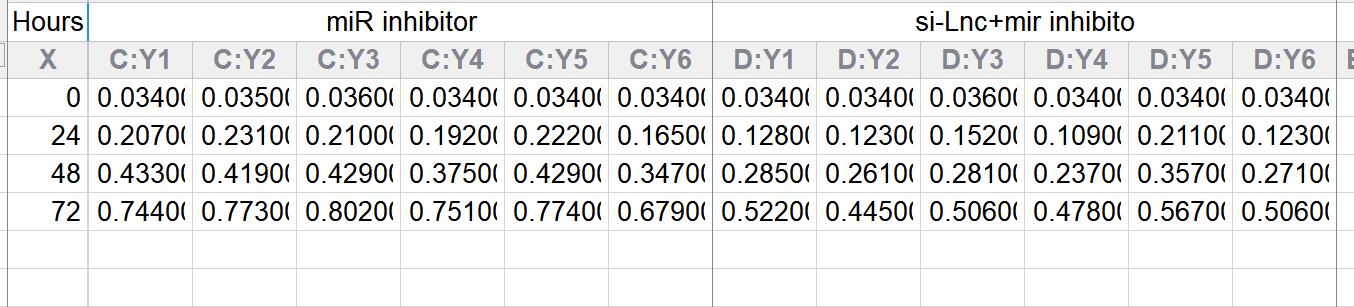

Supplement: Supplemental Information 4 [file peerj-11-16128-s004.zip › date Fig 4/Fig4 date/CCK8 2.png]

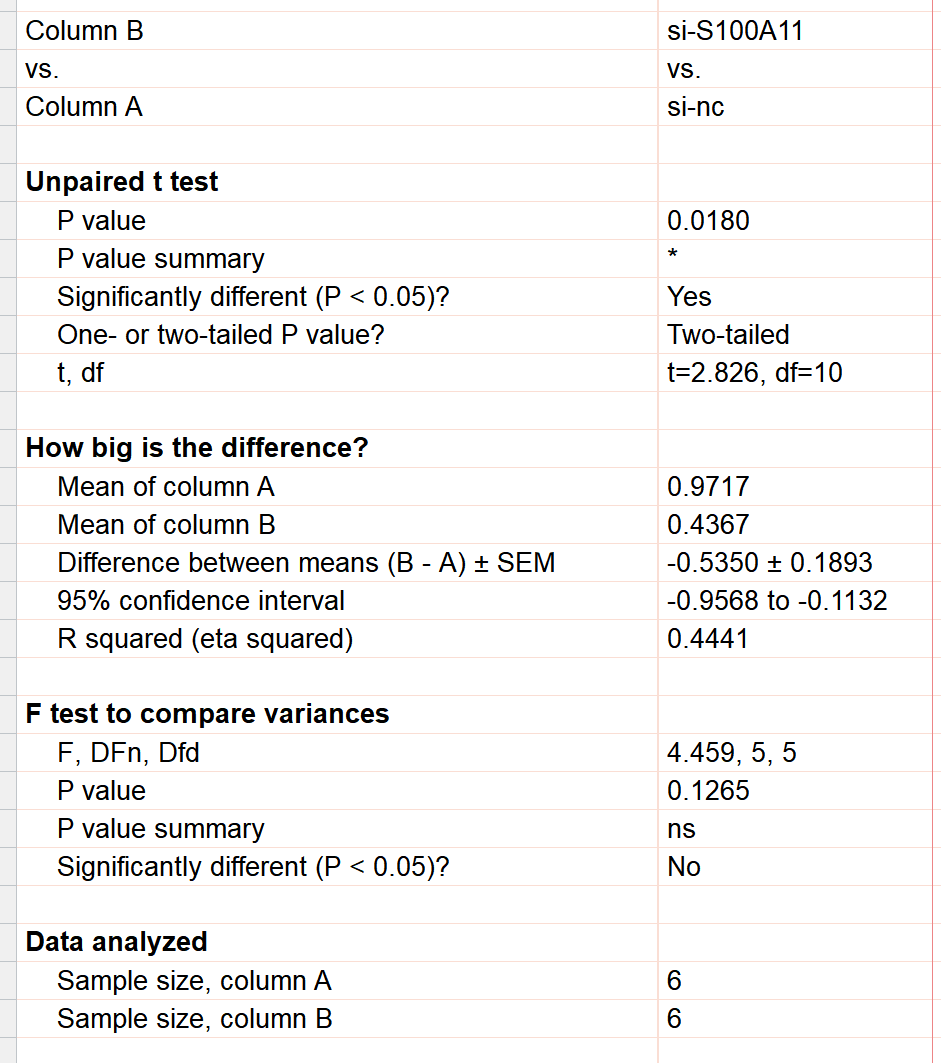

Supplement: Supplemental Information 4 [file peerj-11-16128-s004.zip › date Fig 4/Fig4 date/LEF1-AS1.png]

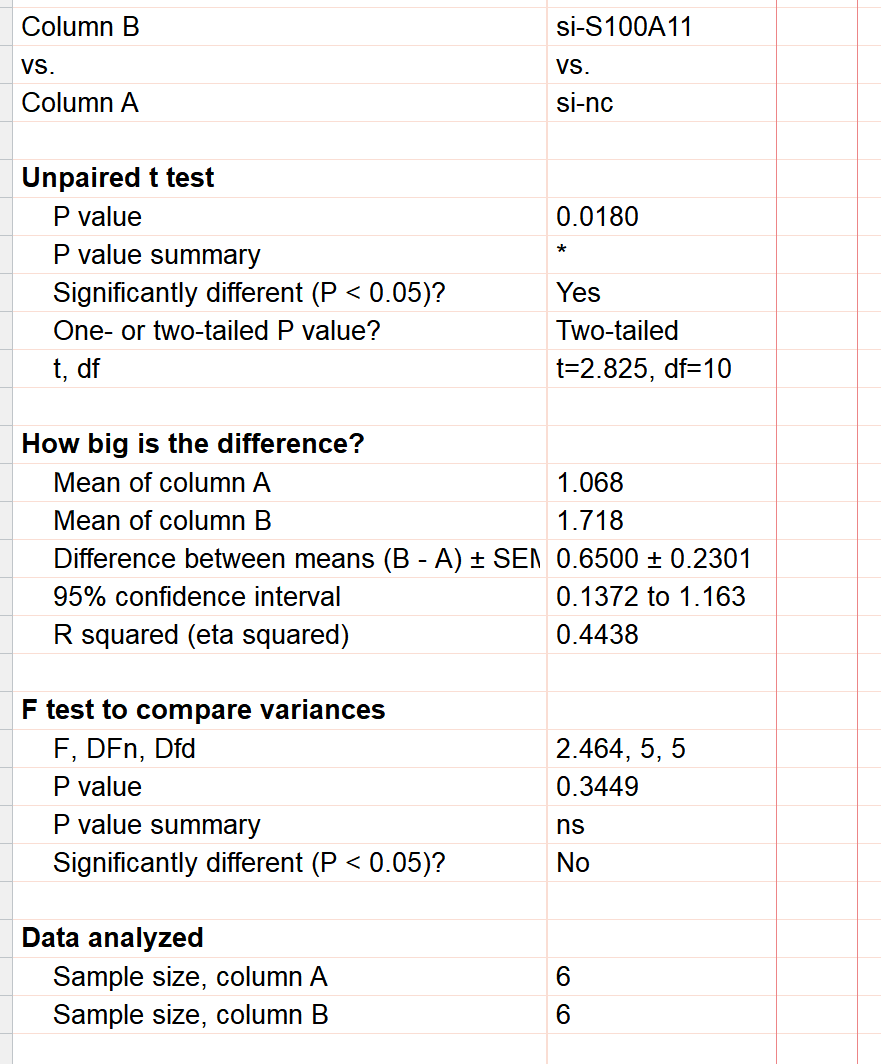

Supplement: Supplemental Information 4 [file peerj-11-16128-s004.zip › date Fig 4/Fig4 date/miR-489-3p.png]

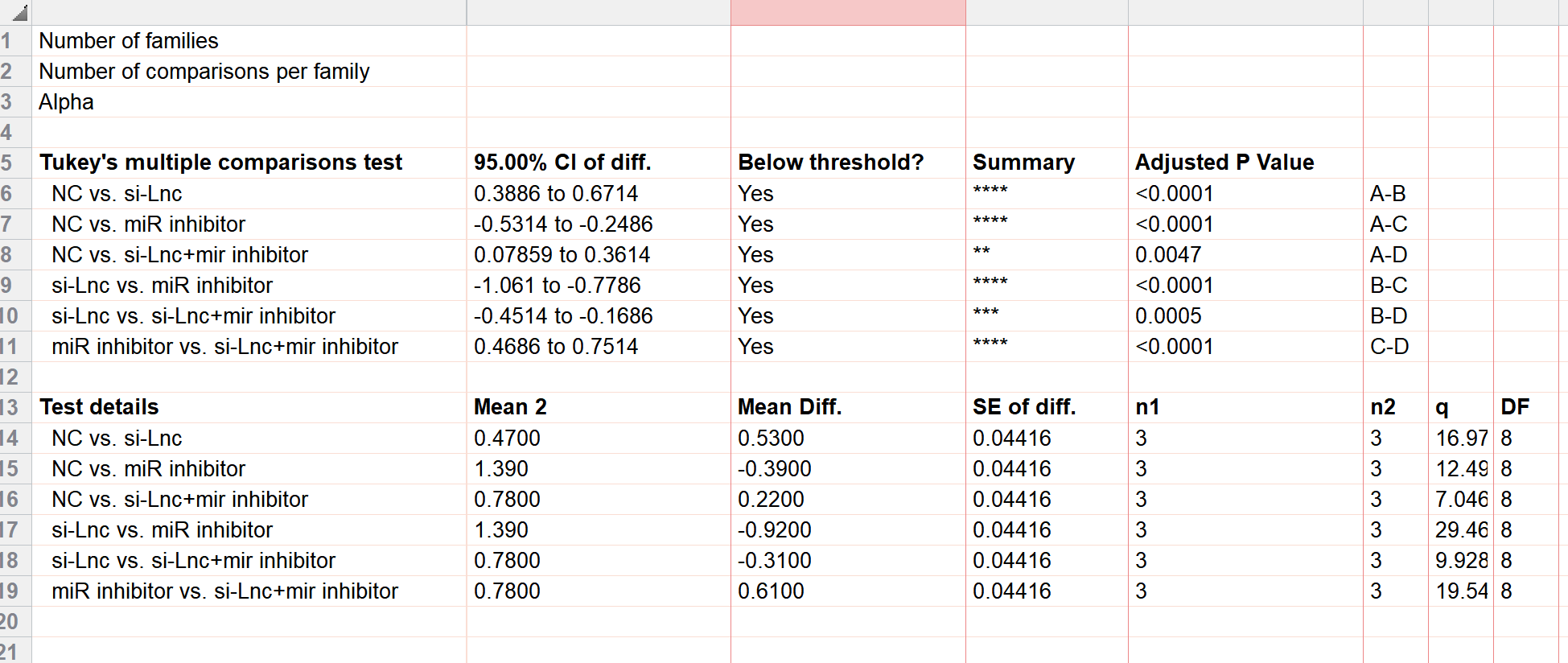

Supplement: Supplemental Information 4 [file peerj-11-16128-s004.zip › date Fig 4/Fig4 date/S100A11.png]

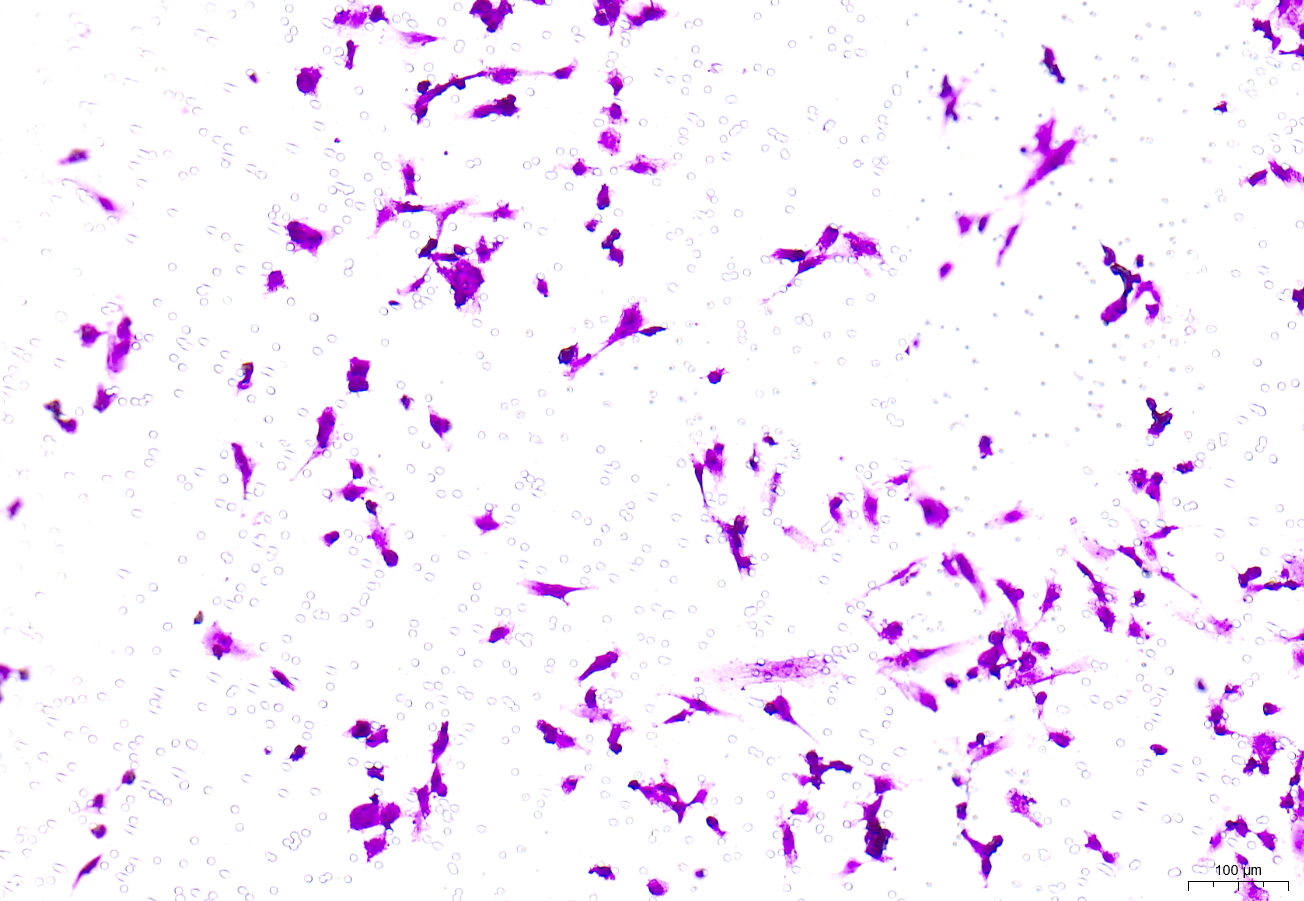

Supplement: Supplemental Information 4 [file peerj-11-16128-s004.zip › date Fig 4/Fig4 Migration/figure/rep1/1:NC组.jpg]

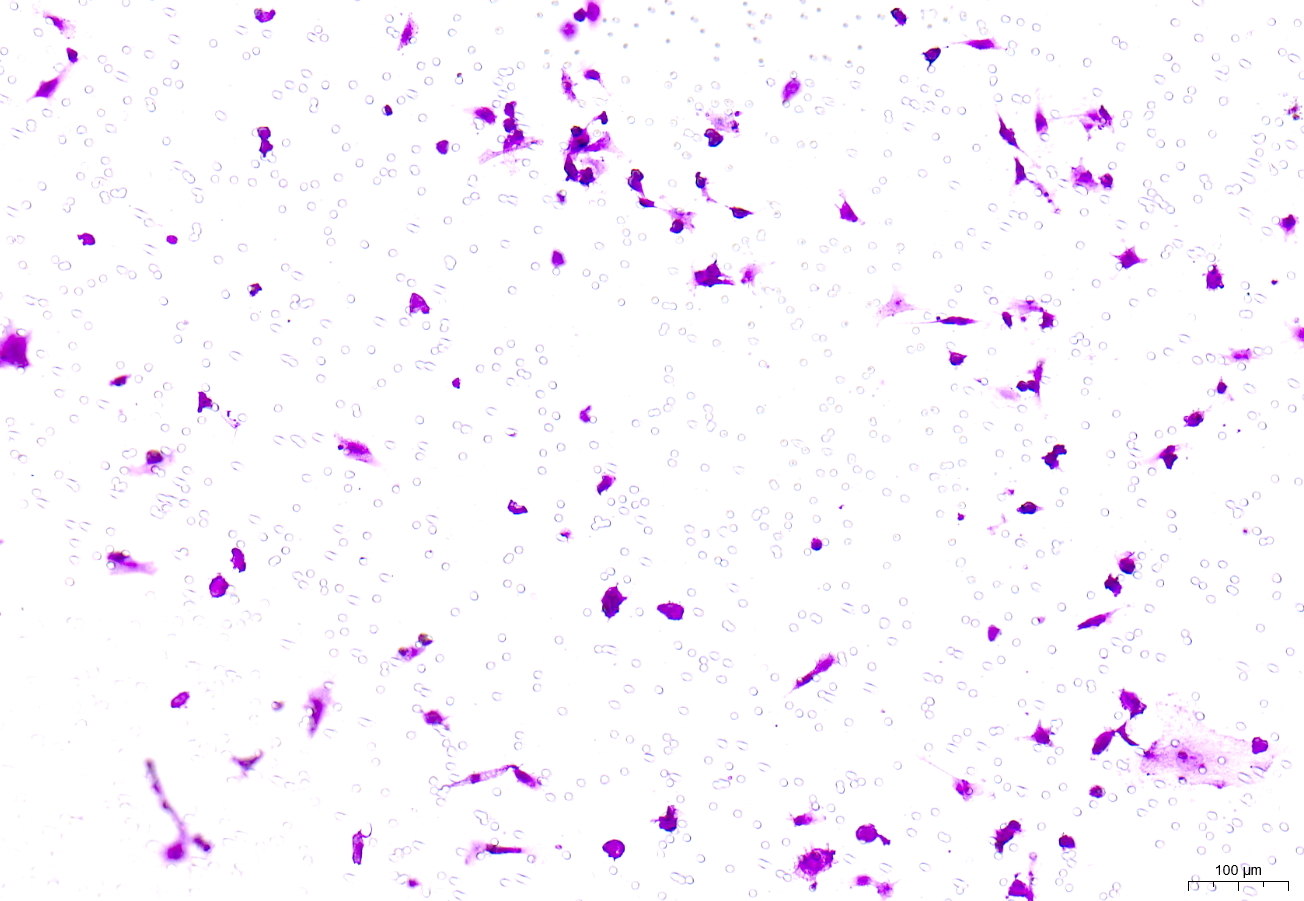

Supplement: Supplemental Information 4 [file peerj-11-16128-s004.zip › date Fig 4/Fig4 Migration/figure/rep1/2:si-Lnc组.jpg]

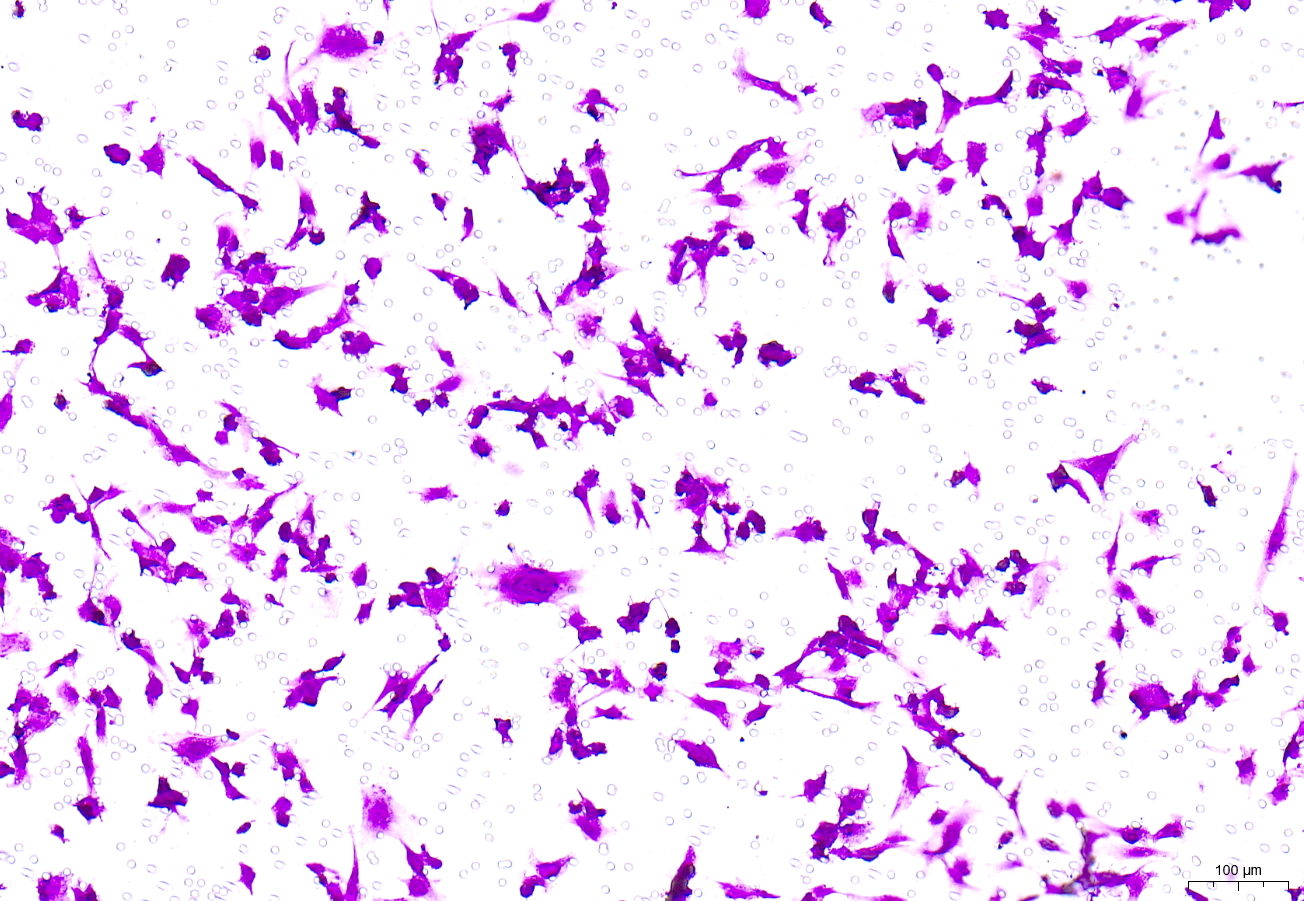

Supplement: Supplemental Information 4 [file peerj-11-16128-s004.zip › date Fig 4/Fig4 Migration/figure/rep1/3:miR inhibitor.jpg]

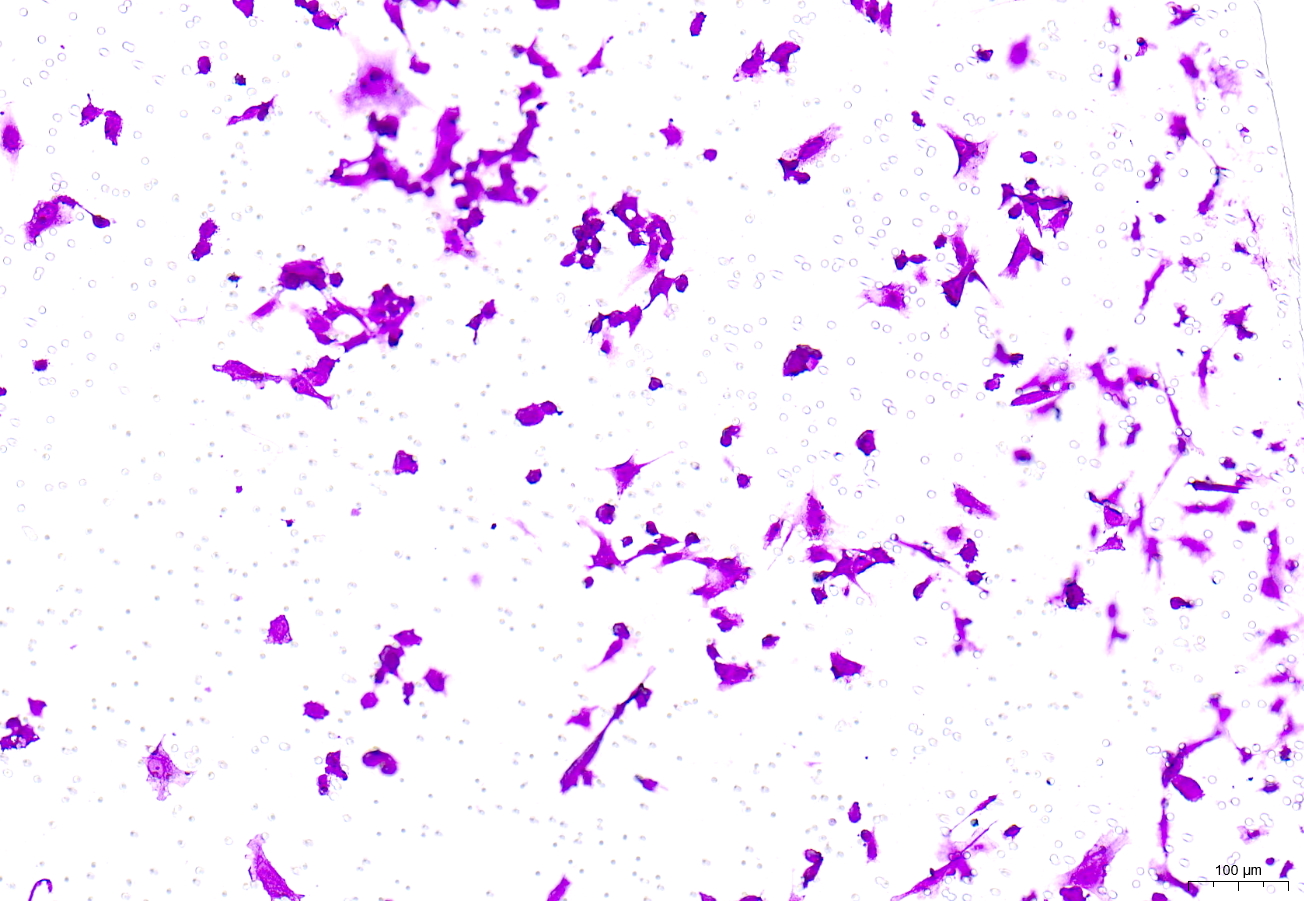

Supplement: Supplemental Information 4 [file peerj-11-16128-s004.zip › date Fig 4/Fig4 Migration/figure/rep1/4:si-Lnc+mir inhibito.jpg]

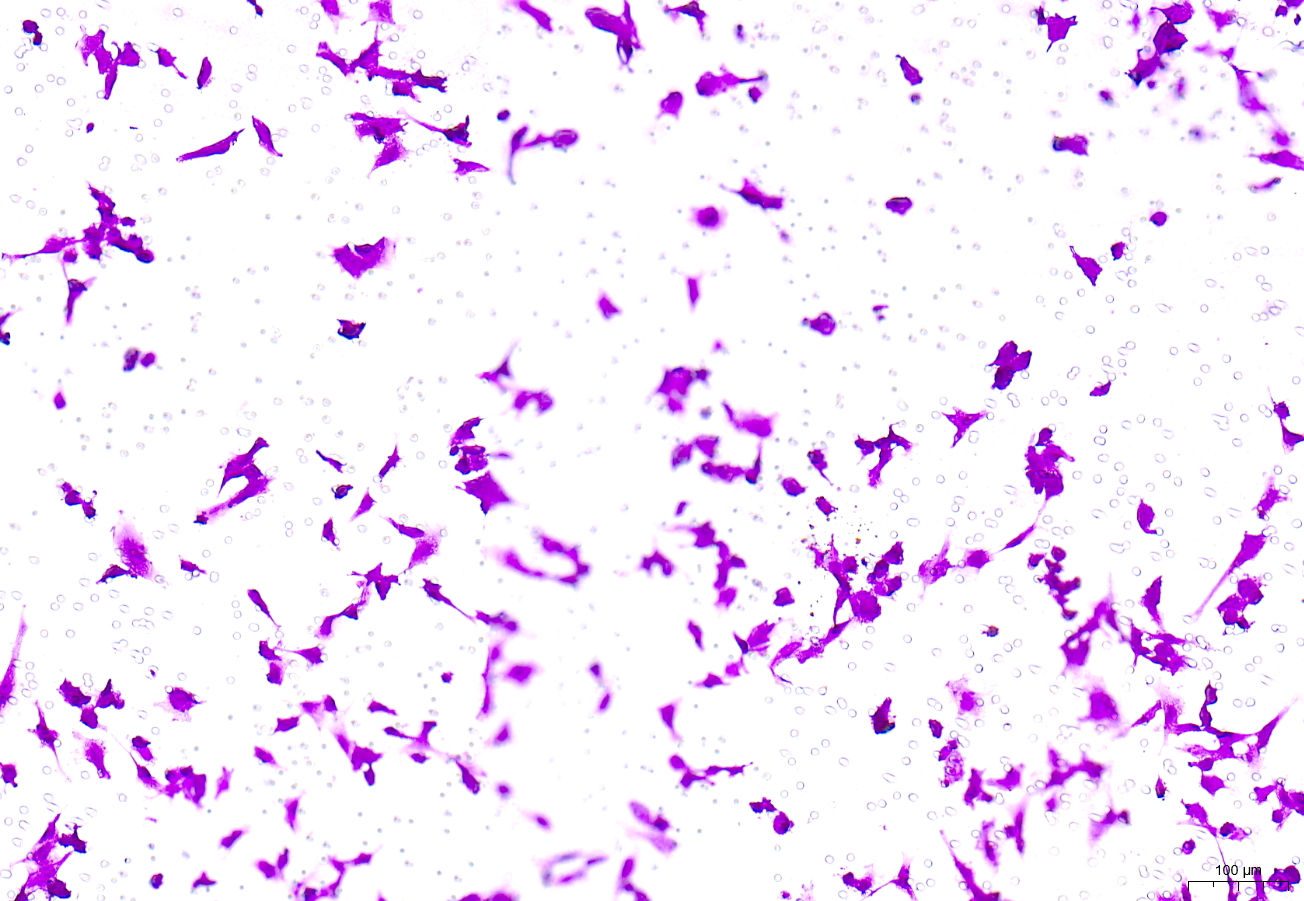

Supplement: Supplemental Information 4 [file peerj-11-16128-s004.zip › date Fig 4/Fig4 Migration/figure/rep2/1:NC组.jpg]

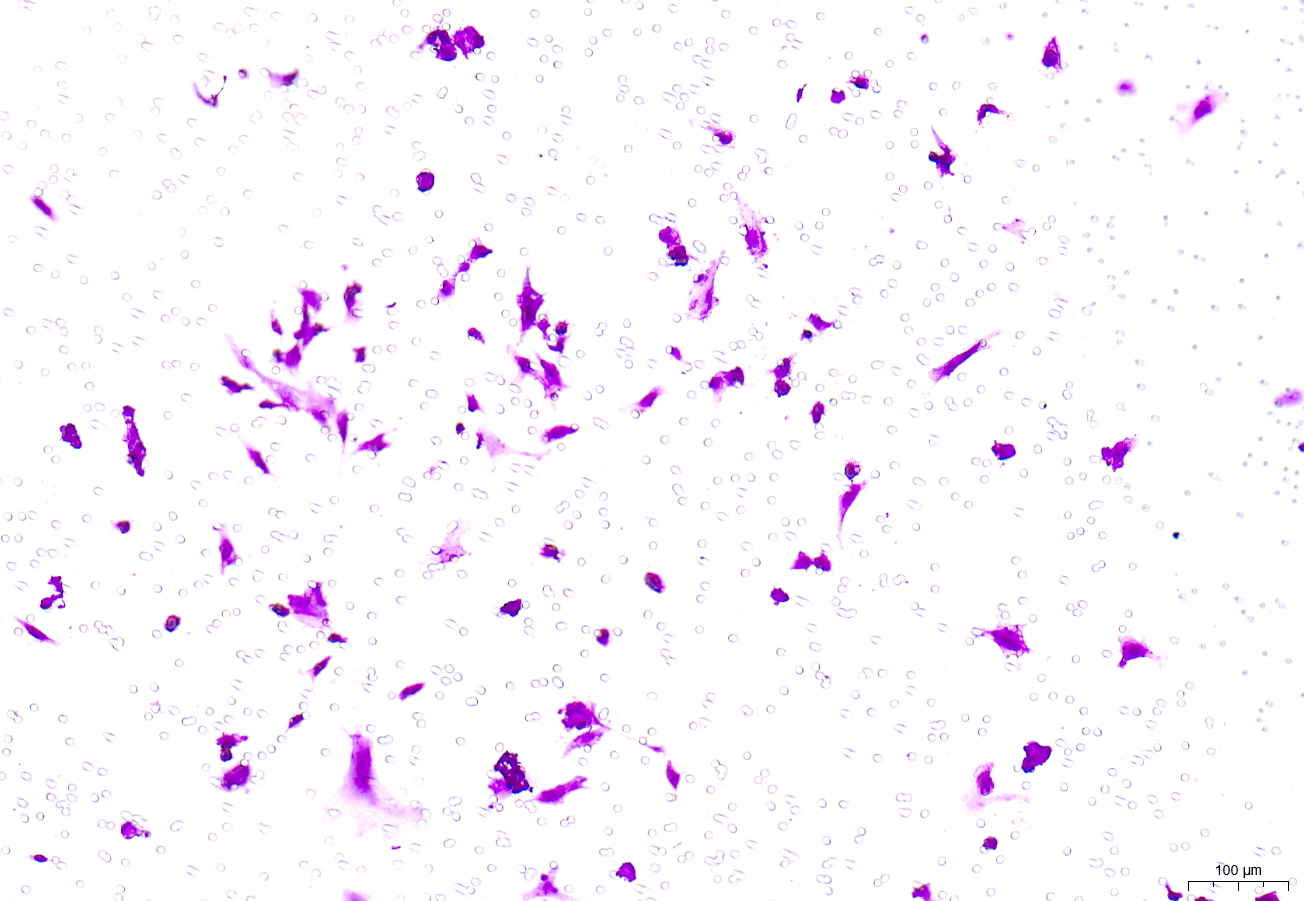

Supplement: Supplemental Information 4 [file peerj-11-16128-s004.zip › date Fig 4/Fig4 Migration/figure/rep2/2:si-Lnc组.jpg]

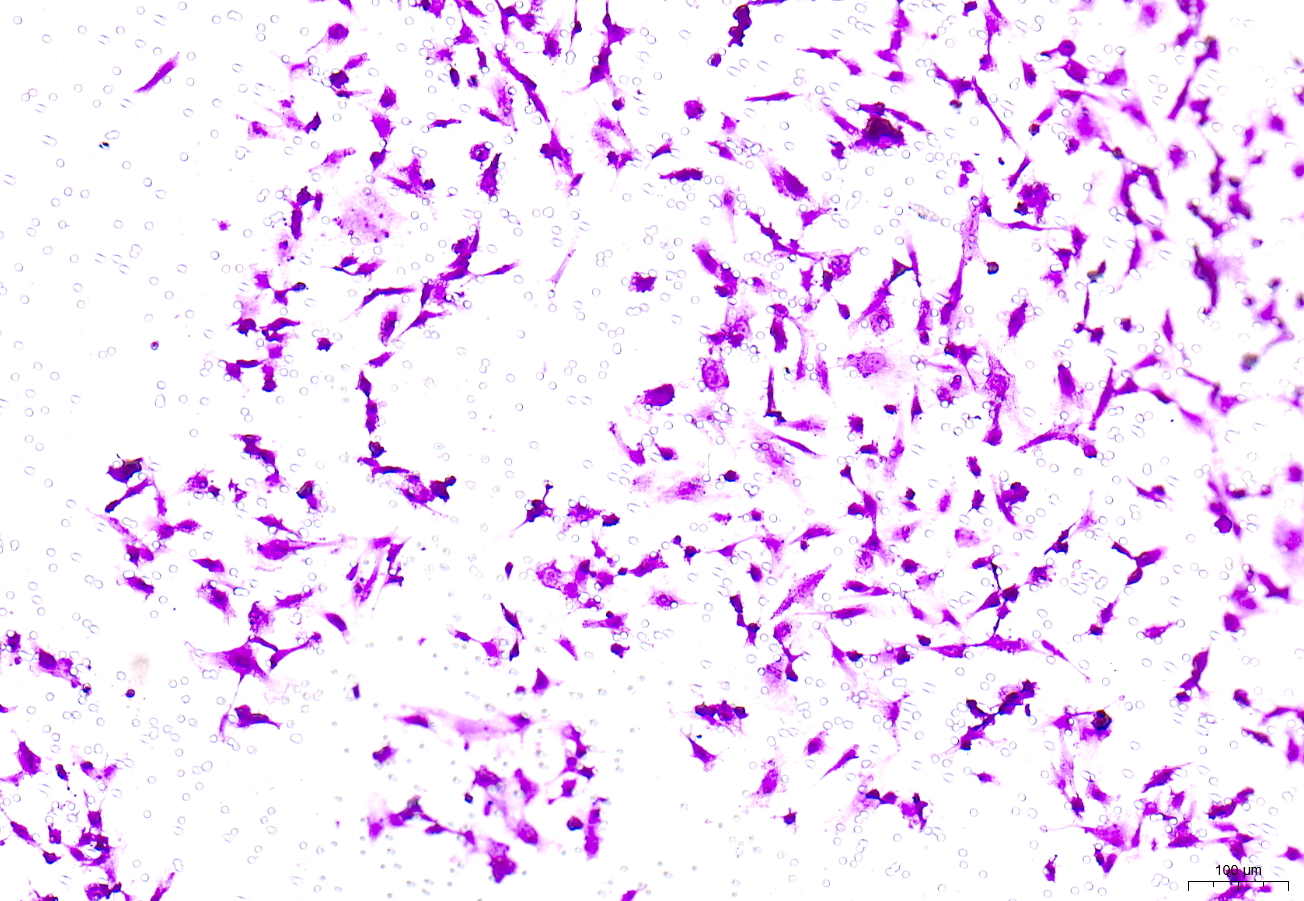

Supplement: Supplemental Information 4 [file peerj-11-16128-s004.zip › date Fig 4/Fig4 Migration/figure/rep2/3:miR inhibitor .jpg]

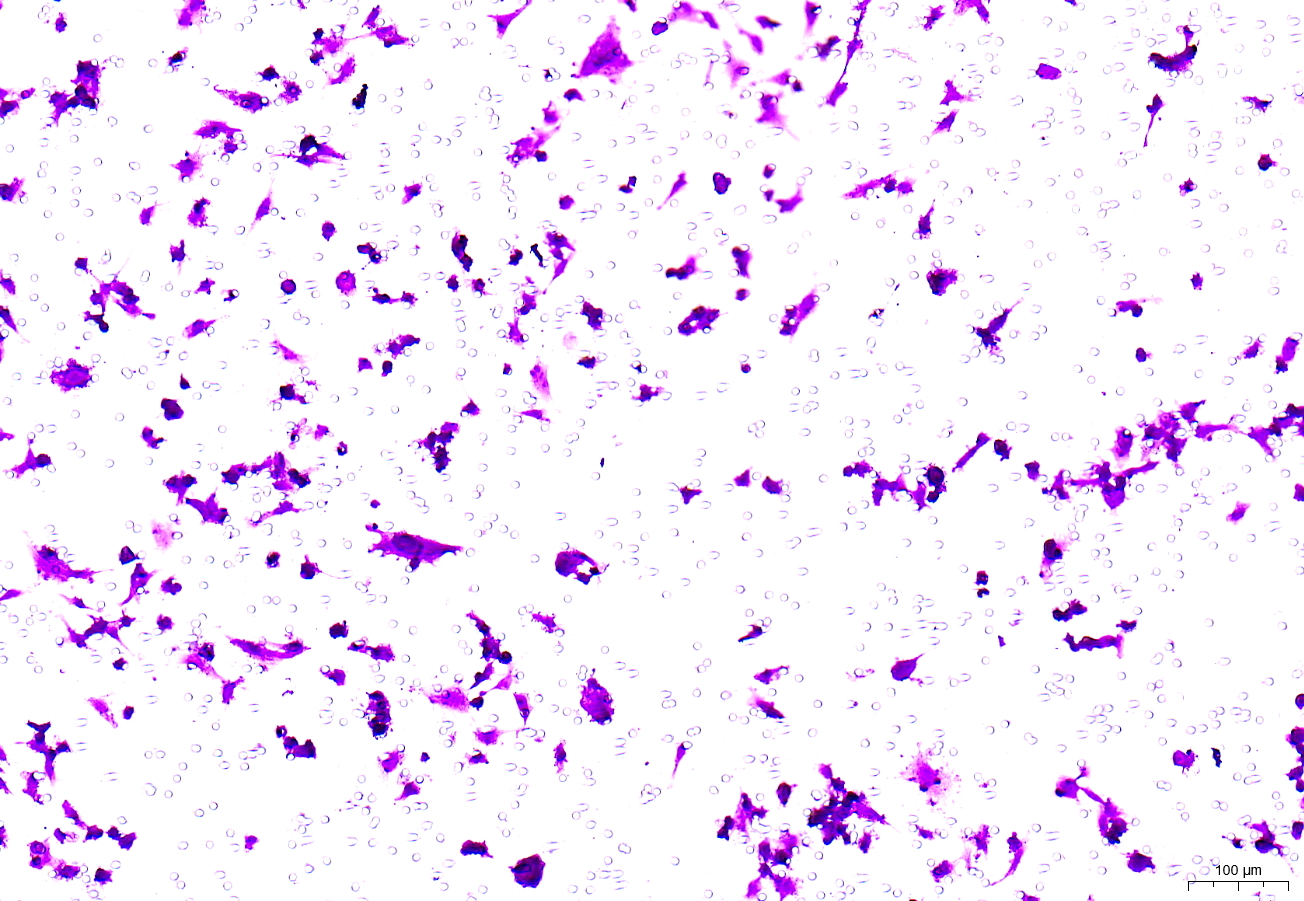

Supplement: Supplemental Information 4 [file peerj-11-16128-s004.zip › date Fig 4/Fig4 Migration/figure/rep2/4:si-Lnc+mir inhibitor.jpg]

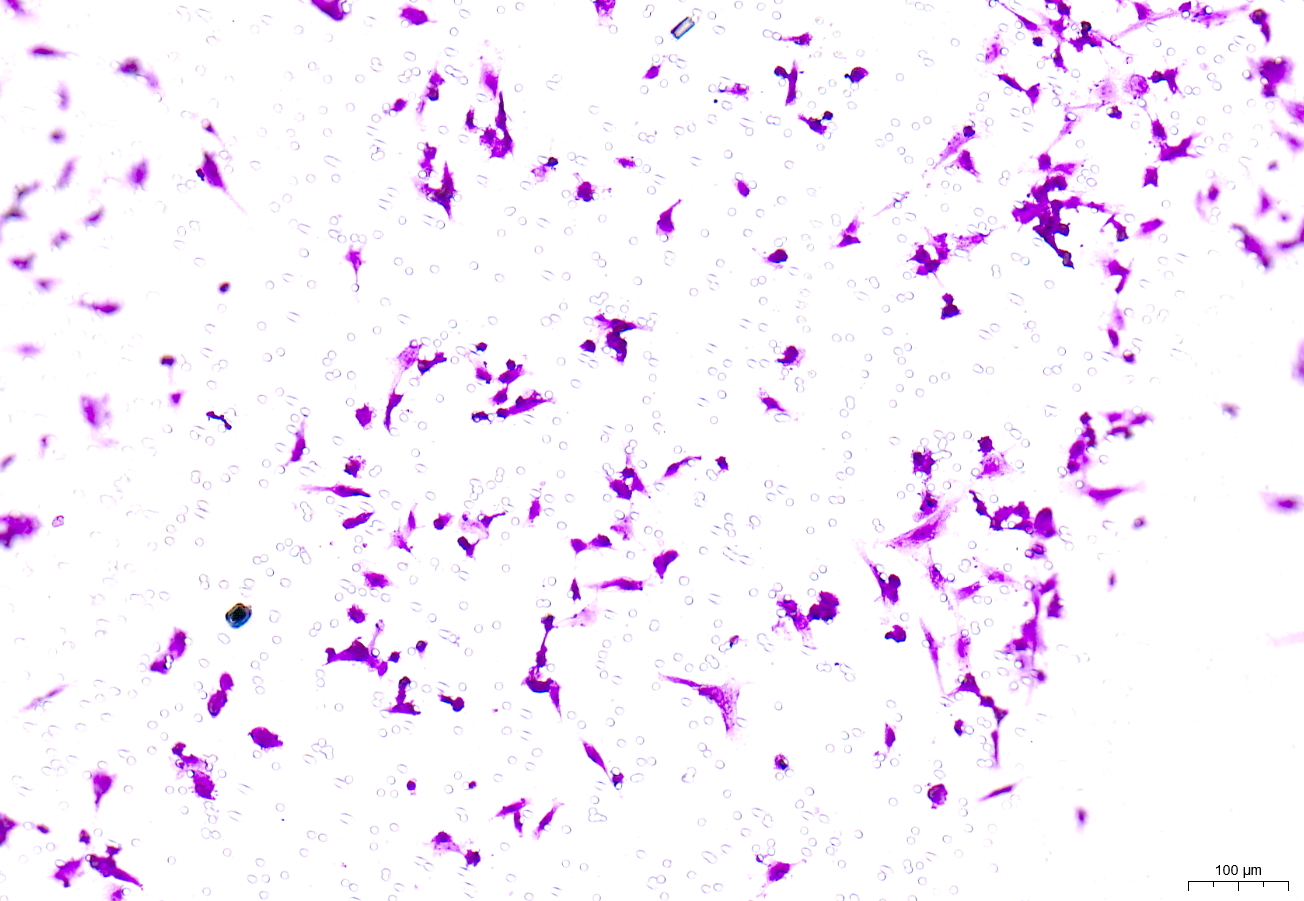

Supplement: Supplemental Information 4 [file peerj-11-16128-s004.zip › date Fig 4/Fig4 Migration/figure/rep3/1:NC组 .jpg]

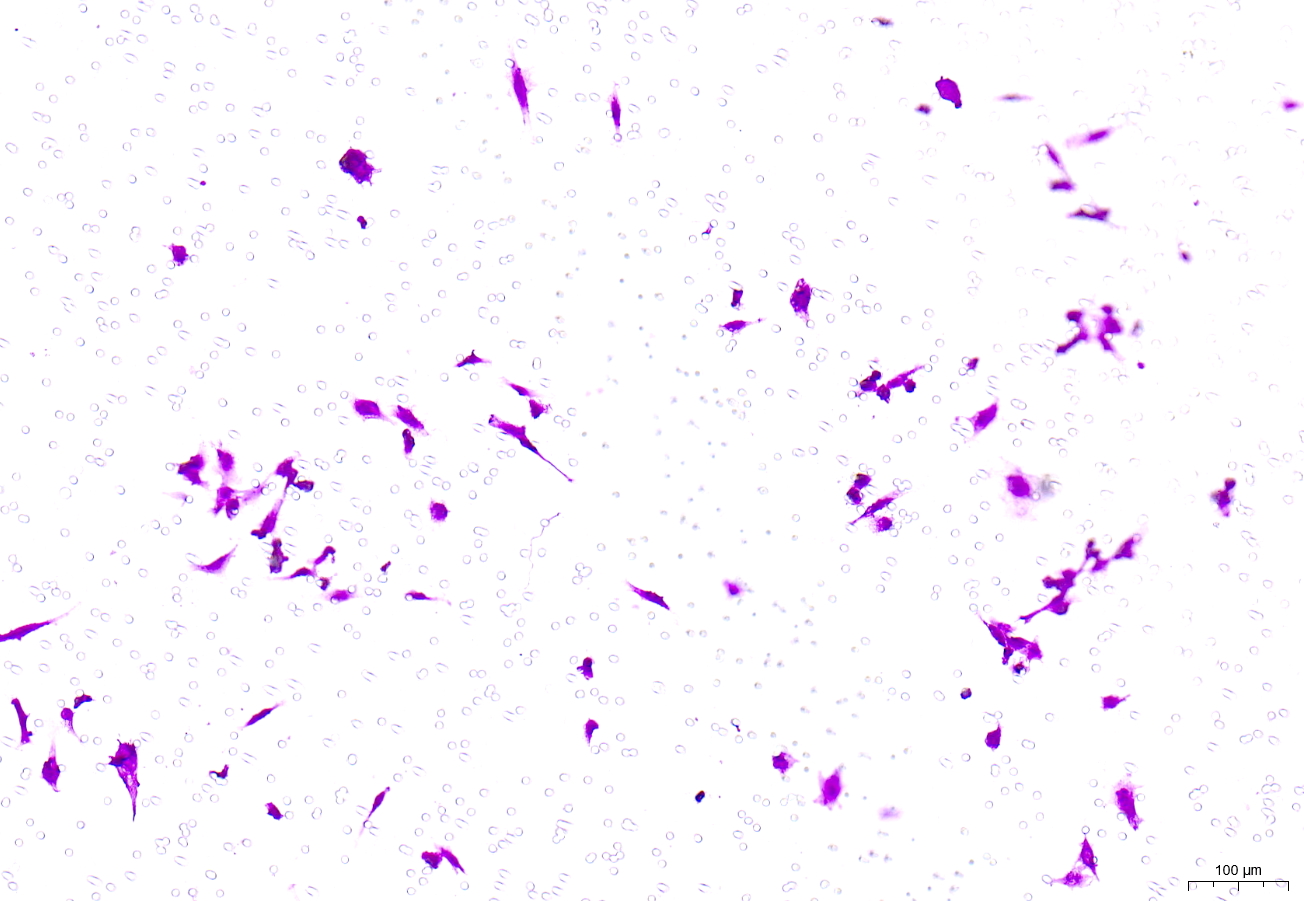

Supplement: Supplemental Information 4 [file peerj-11-16128-s004.zip › date Fig 4/Fig4 Migration/figure/rep3/2:si-Lnc组 .jpg]

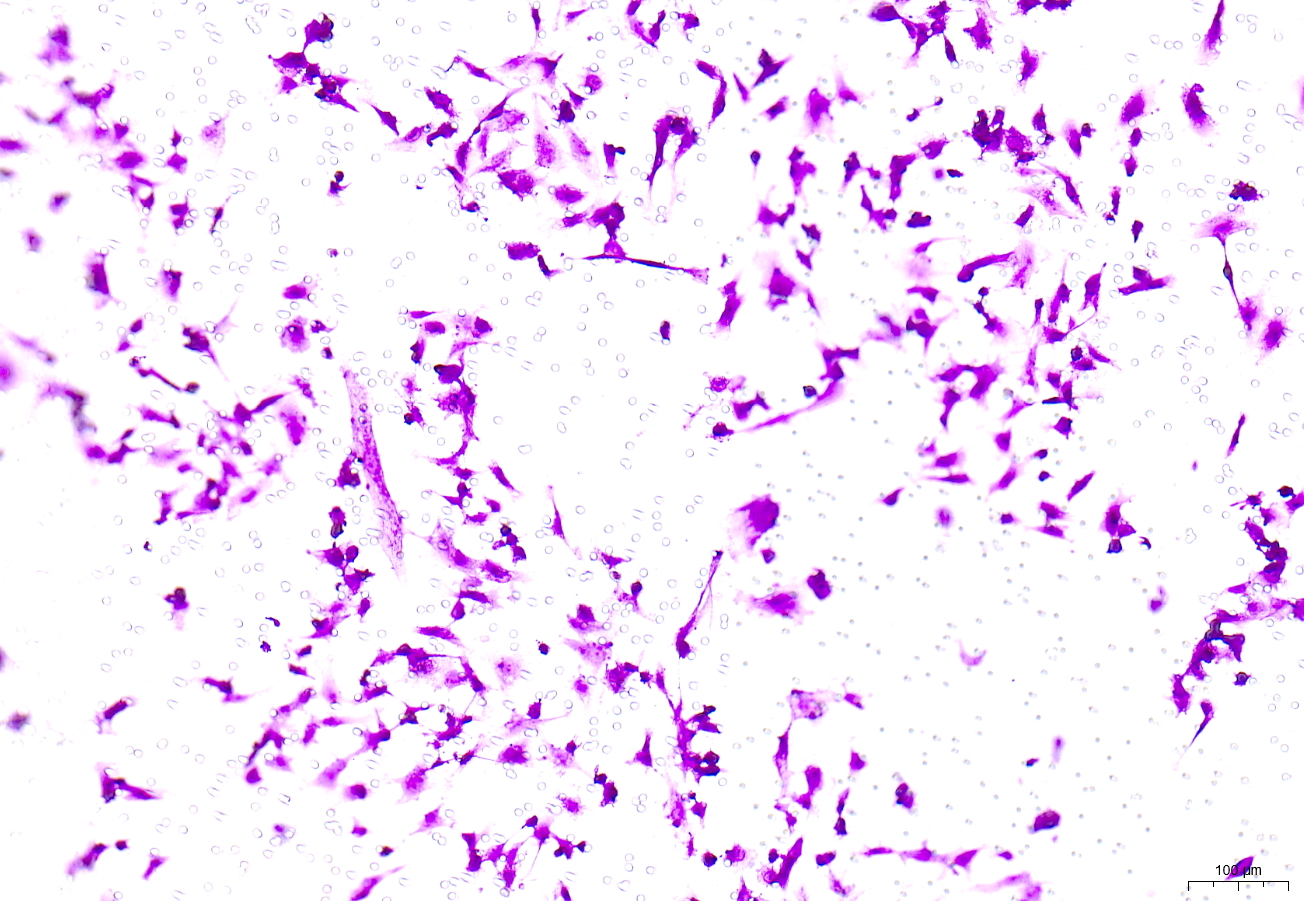

Supplement: Supplemental Information 4 [file peerj-11-16128-s004.zip › date Fig 4/Fig4 Migration/figure/rep3/3:miR inhibitor.jpg]

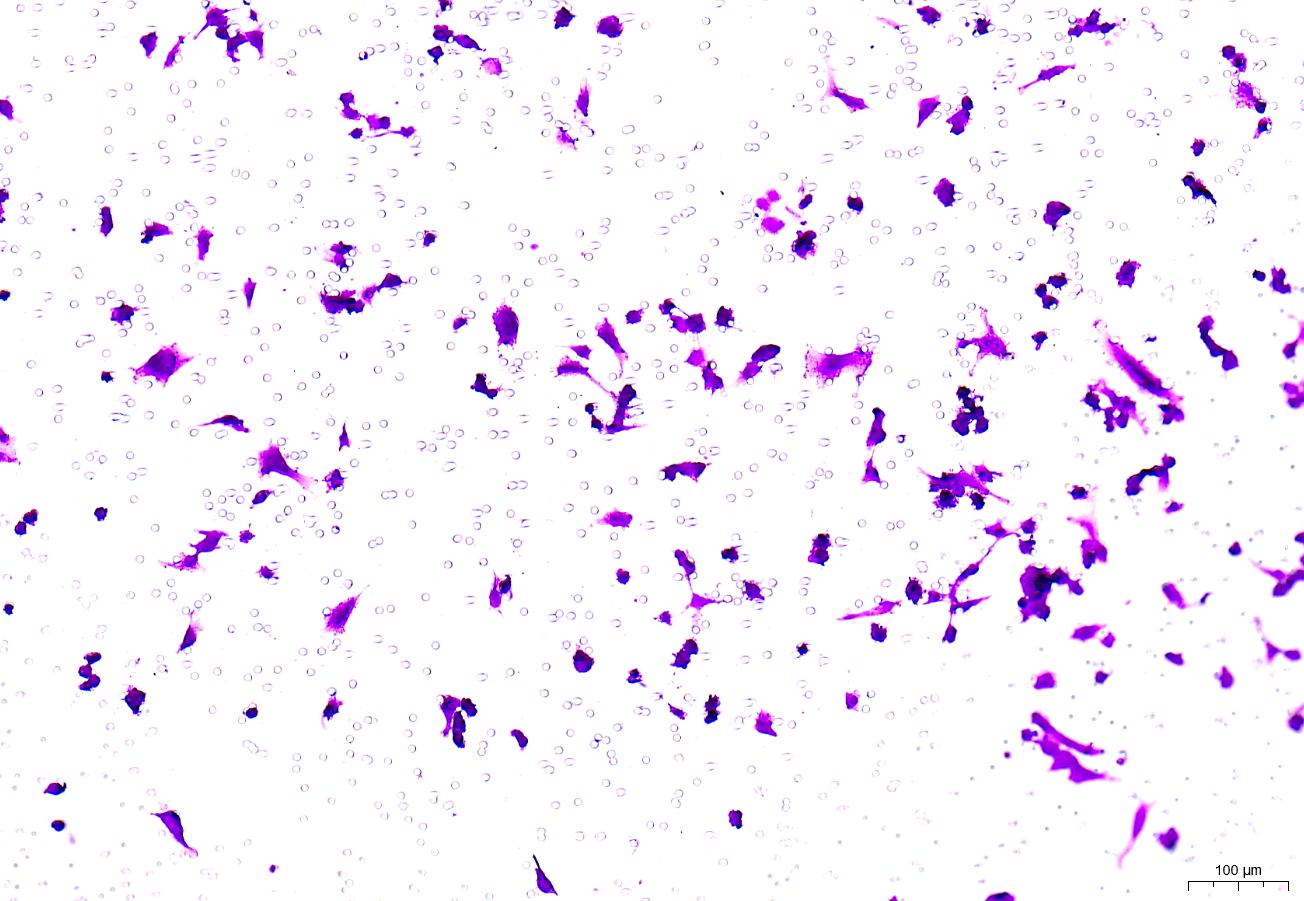

Supplement: Supplemental Information 4 [file peerj-11-16128-s004.zip › date Fig 4/Fig4 Migration/figure/rep3/4:si-Lnc+mir inhibitor.jpg]

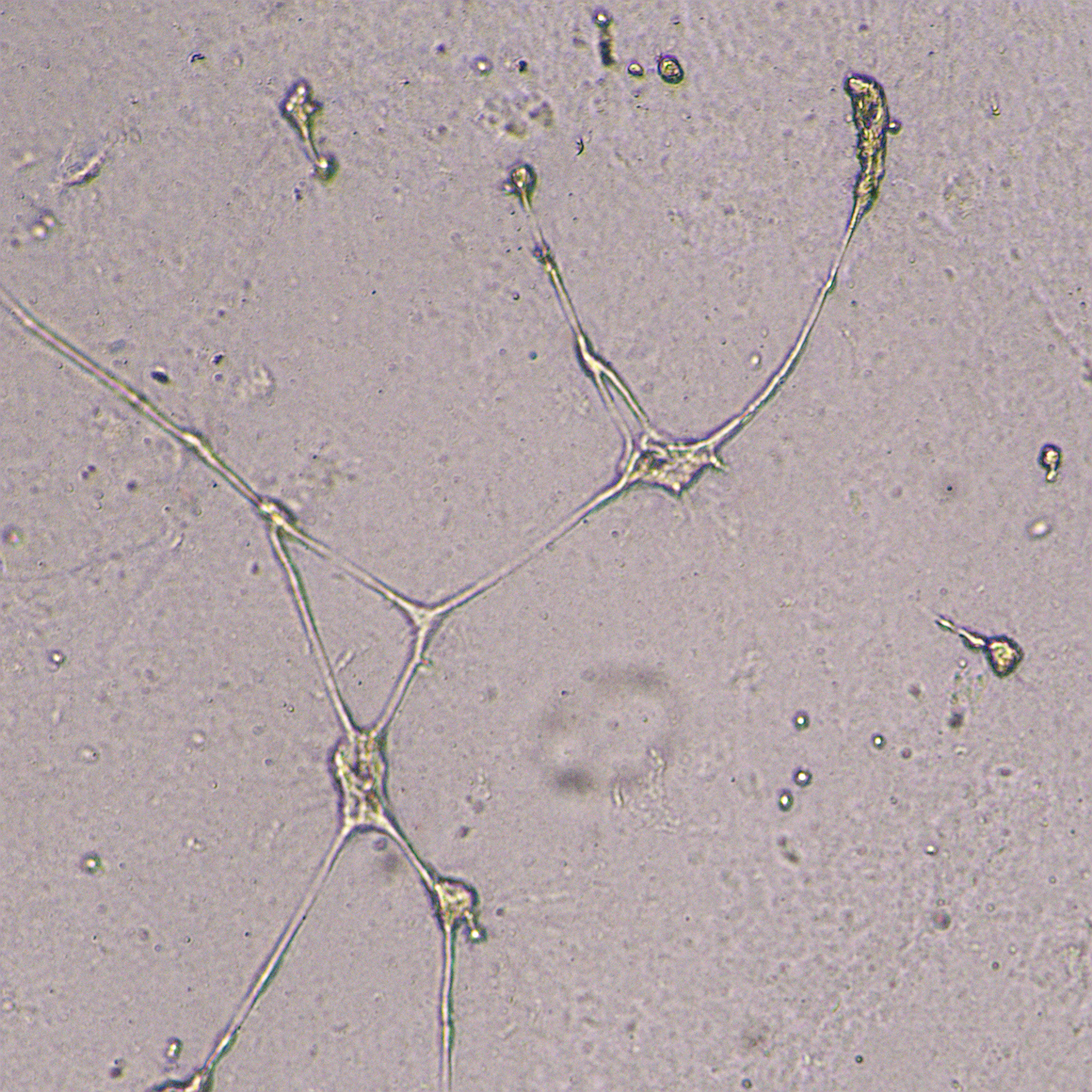

Supplement: Supplemental Information 5 [file peerj-11-16128-s005.zip › Fig4 Angiogenesis SI/SI(1).jpg]

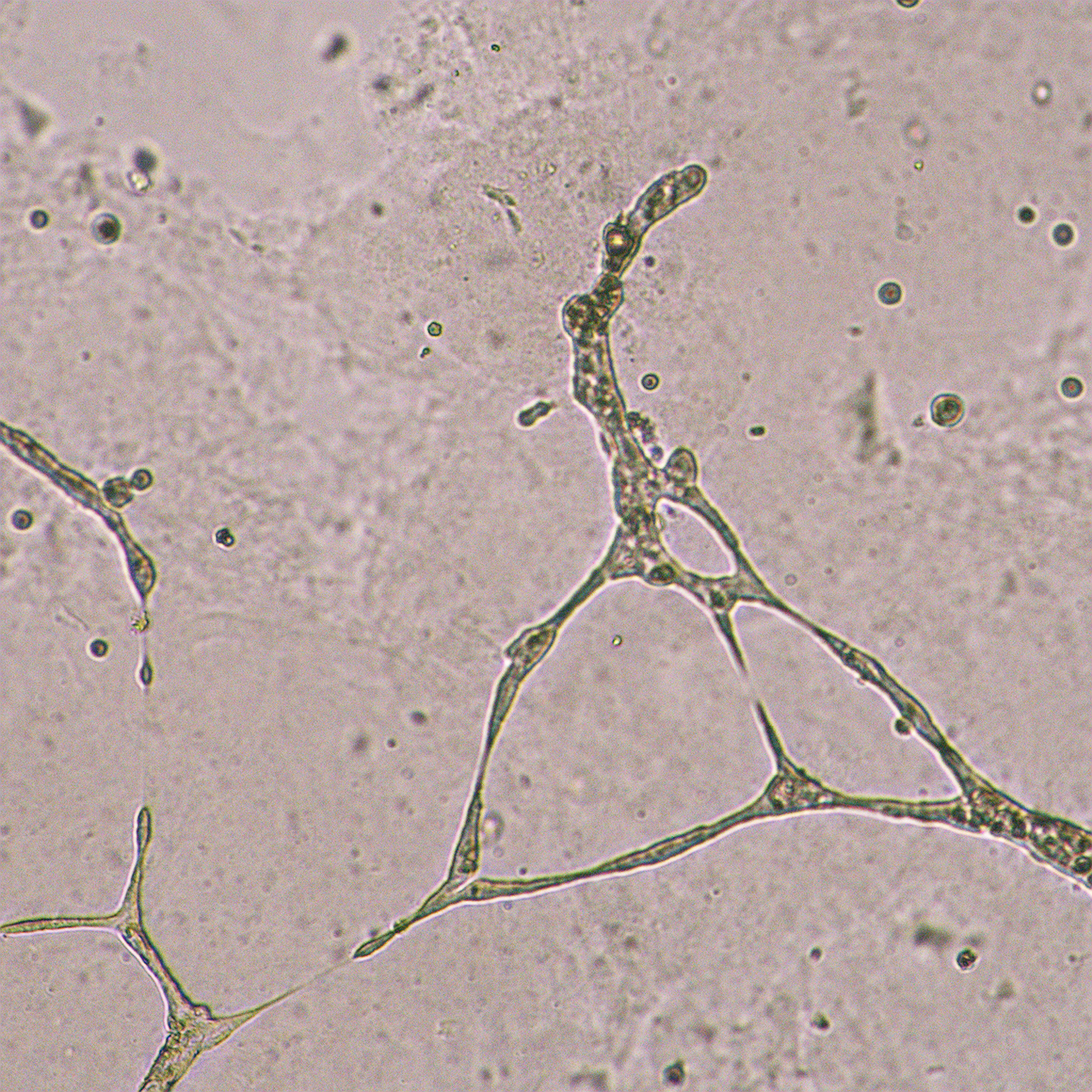

Supplement: Supplemental Information 5 [file peerj-11-16128-s005.zip › Fig4 Angiogenesis SI/SI(2).jpg]

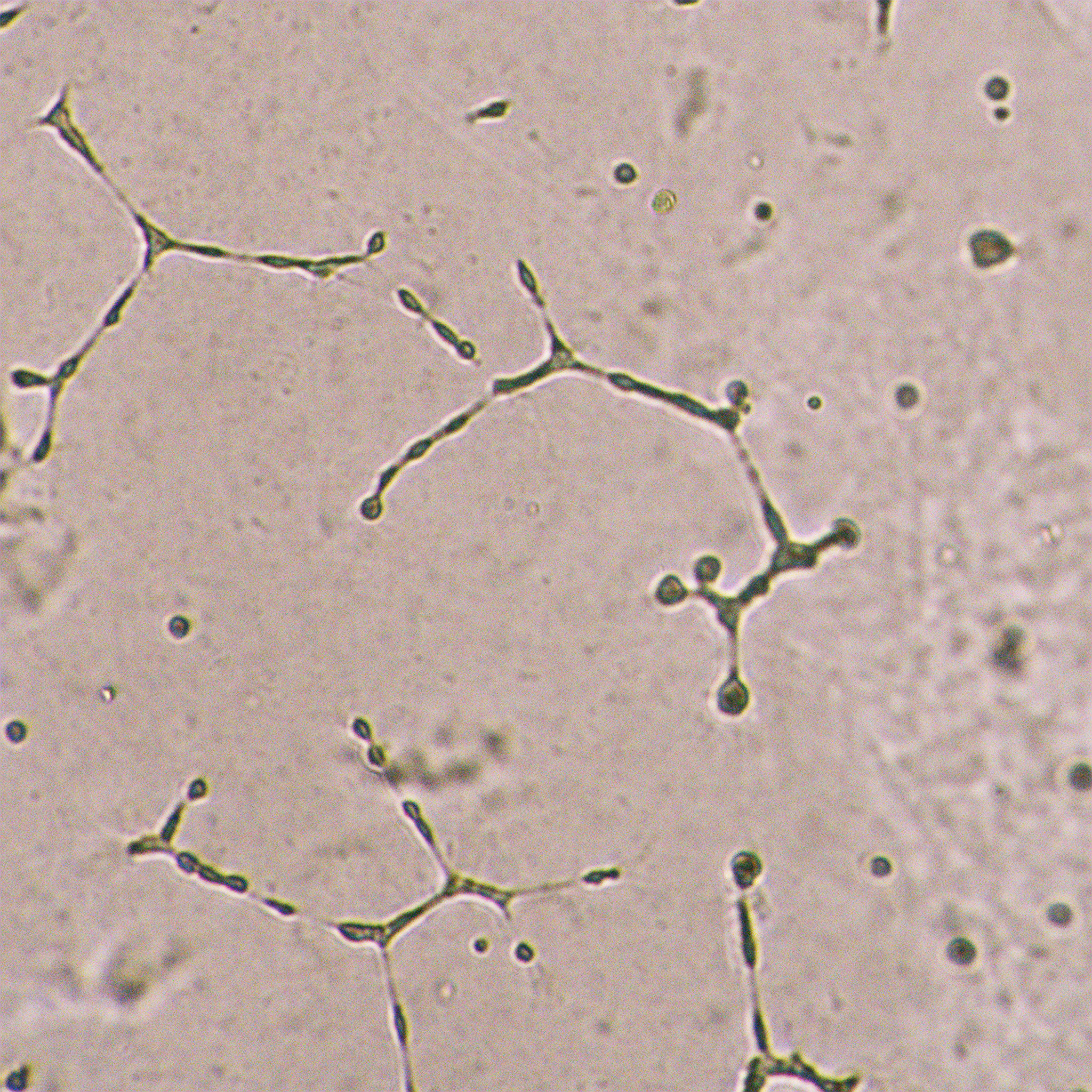

Supplement: Supplemental Information 5 [file peerj-11-16128-s005.zip › Fig4 Angiogenesis SI/SI(3).jpg]
